# CT-derived subcutaneous adipose tissue thickness and circulating adipokines in relation to neurological outcome after in-hospital cardiac arrest: two parallel cohort analyses

**DOI:** 10.3389/fneur.2026.1898760

**Published:** 2026-10-20

**Authors:** Tingyuan Zhou, Wen Ma, Yarong He, Songling Tang, Peng Yao, Lu Gan, Yu Cao

**Affiliations:** 1 Sichuan University-Hong Kong Polytechnic University Institute for Disaster Management and Reconstruction, Chengdu, China; 2 Department of Emergency Medicine, Institute of Disaster Medicine and Institute of Emergency Medicine, West China Hospital, Sichuan University, Chengdu, China

**Keywords:** adipokine, in-hospital cardiac arrest, neurological outcome, subcutaneous adipose tissue, thoracic cage

## Abstract

**Background:**

Hypoxic–ischemic brain injury is a major determinant of outcome after in-hospital cardiac arrest (IHCA). Adipose tissue may be relevant to post-arrest outcomes through its anatomical and endocrine characteristics. We conducted two parallel exploratory analyses to examine the associations of thoracic subcutaneous adipose tissue depth and circulating adipokines with neurological outcome after IHCA.

**Method:**

We employed a dual-cohort design. The CT Imaging Cohort included patients with pre-arrest and post-arrest thoracic CT examinations. Associations of anterior and posterior subcutaneous adipose tissue thickness with survival and neurological outcome at hospital discharge were evaluated. In the Biochemical Cohort, plasma concentrations of 12 adipokines measured during cardiac arrest were compared between neurological outcome groups. Multiple comparisons were controlled using the Benjamini–Hochberg false discovery rate procedure. Exploratory logistic regression models were evaluated using discrimination, calibration, bootstrap internal validation, and Firth regression sensitivity analysis.

**Results:**

In the CT Imaging Cohort, several unadjusted differences in post-arrest posterior subcutaneous adipose tissue thickness were observed between neurological outcome groups, but none remained statistically significant after false discovery rate correction in the overall analysis. In the ventilation-matched sensitivity analysis, greater pSAT thickness at selected thoracic levels remained associated with favorable neurological outcome after correction for multiple comparisons. In the Biochemical Cohort, CTRP6, CTRP9, Omentin-1, and Vaspin concentrations were higher and Chemerin concentration was lower in patients with favorable neurological outcomes after false discovery rate correction. The apparent AUC was 0.876 (95% CI, 0.795–0.957) for the clinical model and 0.938 (95% CI, 0.882–0.993) for the exploratory model adding CTRP6. The AUC difference was not statistically significant (ΔAUC, 0.062; 95% CI, −0.003 to 0.126; *P* = 0.062). The optimism-corrected AUC of the extended model was 0.930.

**Conclusion:**

CT-derived posterior subcutaneous adipose tissue thickness and circulating adipokines showed exploratory associations with neurological outcome in two independent IHCA cohorts. The addition of CTRP6 to CPR duration and lactate numerically improved model discrimination, but the incremental AUC was not statistically significant. These findings are hypothesis-generating and require external validation. Because CT measurements and adipokines were obtained from different patients, the study does not establish a direct anatomical–biochemical relationship.

## Introduction

In-hospital cardiac arrest (IHCA) is a catastrophic clinical event, with survivors frequently suffering from neurological deficits driven by acute hypoxic-ischemic brain injury (HIBI) (1). Following the initial ischemic insult and the subsequent return of spontaneous circulation (ROSC), patients experience ischemia-reperfusion syndrome characterized by secondary neuroinflammatory cascade (2). These systemic and neurological responses may contribute to secondary tissue injury and functional outcomes. Despite advances in post-resuscitation care, accurately predicting early neurological recovery remains a major clinical challenge (2). Current prognostic modalities often lack sufficient sensitivity or are confounded using sedatives and targeted temperature management. Therefore, additional objective markers may help characterize the risk of unfavorable neurological outcome after IHCA.

Chest compression is at the core of cardiopulmonary resuscitation (CPR) (3, 4). According to the cardiac pump theory, external chest compressions directly compress the heart between the sternum and vertebral column, thereby contributing to forward blood flow (5–8). During this procedure, subcutaneous adipose tissue (SAT) and the bony thorax lie along the pathway through which external compression forces are transmitted to the thorax (9, 10). Previous studies have suggested that thoracic dimensions and SAT thickness may influence the effectiveness of chest compressions and clinical outcomes (9). Paradoxically, in some meta-analyses, moderately overweight cardiac arrest (CA) patients generally have more favorable neurological outcomes (11–13). However, in obese CA patients CC efficacy could be undermined (10).

While the aforementioned “obese paradox” has been clinically observed in survivors of IHCA, the transition from anatomical fat distribution to molecular neuroprotection remains largely uncharacterized. Traditionally, the bony thorax and SAT have been viewed primarily through a biomechanical lens during CPR, acting merely as physical interfaces for chest compressions. This mechanical perspective has often been invoked to debate the controversial “obesity paradox,” wherein moderately overweight CA patients paradoxically exhibit more favorable neurological outcomes (14). Emerging evidence suggests that specific adipose depots, such as SAT, may act as surrogates for the body's endocrine capacity to secrete neuroprotective adipokines—including CTRP9, Omentin-1, CTRP12, and Vaspin—which have suggested potent anti-apoptotic and anti-inflammatory effects in ischemic brain injury models (15–17).

We hypothesized that CT-derived SAT thickness and circulating adipokine profiles may be associated with neurological outcomes after IHCA. To explore this hypothesis, we conducted two conceptually sequential but analytically independent exploratory investigations in separate cohorts. First, the CT Imaging Cohort was used to examine associations between CT-derived SAT thickness before and after CA and patient outcomes. Motivated by the exploratory anatomical observations, circulating adipokine profiles measured at the time of CA were subsequently evaluated in an independent Biochemical Cohort in relation to neurological outcomes. These analyses were intended to provide complementary anatomical and biochemical observations and to generate hypotheses regarding adipose-related factors in neurological recovery after IHCA.

## Methods

### Study design and setting

The study comprised two independent cohorts: a CT Imaging Cohort for exploratory anatomical assessment. Observations from this imaging analysis motivated a subsequent evaluation of circulating adipokines measured at the time of CA in the independent Biochemical Cohort. No participant-level integration of CT and adipokine measurements was performed because the two modalities were assessed in different cohorts.

This study complied with the Declaration of Helsinki and was approved by the Human Ethical Committee of West China Hospital (Reference No. 2021, 1557), which waived the requirement for informed consent due to its observational and retrospective nature.

### Participants

We screened in our electronic medical record (EMR) system for patients with a documented medical order for “cardiopulmonary resuscitation (CPR).”

CT Imaging Cohort (*n* = 70): Adult patients (≥18 years old) who achieved sustained ROSC between May 31, 2019, to June 30, 2021, were eligible. Inclusion required available enhanced or non-enhanced CT scans both before and after cardiopulmonary resuscitation (CPR). Exclusion criteria included: (1) severe thoracic trauma or deformity; (2) prior thoracotomy; (3) inconsistent posture between CT scans; (4) pregnancy or lactation; or (5) incomplete medical records.

Biochemical Cohort (*n* = 66): A separate cohort of patients with cardiac arrest was enrolled between Jul 1, 2021, to Feb 28, 2022. Inclusion required the availability of plasma samples collected at the time of CA. The exclusion criteria included: (1) Out-of-hospital cardiac arrest (OHCA); (2) Neurogenic cardiac arrest (e.g., arrest secondary to stroke or subarachnoid hemorrhage); (3) Traumatic CA; (4) Hematological malignancies; (5) End-stage chronic diseases (e.g., terminal organ failure);(6) Cachexia (to avoid confounding effects of systemic wasting on adipose measurements); (7) Pregnancy or lactation.

IHCA was defined as cardiac arrest occurring after hospital arrival and requiring chest compressions and/or defibrillation. Patients whose cardiac arrest occurred before hospital arrival were classified as having OHCA and were excluded.

The location of cardiac arrest was classified as the emergency department, intensive care unit, general ward, procedural area, or other in-hospital location, according to the medical record.

Patients who achieved ROSC received post-cardiac arrest care according to institutional practice. However, specific information regarding targeted temperature management, including target temperature, treatment duration, and cooling method, was not consistently recorded and could not be analyzed separately. Data on extracorporeal CPR and detailed targeted temperature management protocols and systematically recorded withdrawal of life-sustaining treatment (WLST) frequency and timing were unavailable, which limited adjustment for differences in post-arrest treatment.

### Outcomes

The outcomes of interest were survival and favorable neurological outcomes at hospital discharge. Definitions of the outcomes were based on the Utstein template. Neurological outcomes were assessed using the cerebral performance category (CPC) scale (18). Patients with CPC scores of 1–2 were classified as having a favorable neurological outcome, whereas those with CPC scores of 3–5 were classified as having an unfavorable neurological outcome.

### Data collection

Patient characteristics, baseline data, timepoints of each CT scan and endotracheal intubation, and data related to CA event were collected using EMR and event protocols. Multidetector CT examinations were performed with a Philips Brilliance 16-slice MDCT (Philips Healthcare, Cleveland, Ohio) with a 5-mm slice thickness. Unenhanced cross-sectional CT images were then analyzed using three-dimensional visualization software (Mimics Interactive Medical Image Control System, Version 21.0; Materialize Company, Belgium). All the images were imported into the software, and the patient's thoracic cage was reconstructed to accurately localize the T3–T12 vertebra and spinous process for further measurement (Supplementary file 2, Supplementary Figure S1). All the included patients had CT images thus there was no missing variables in our study.

Thoracic cage dimension indices, including the transverse diameter (TD) (the shortest distance between the sternal notch and vertebrae), internal anteroposterior diameter (AD) (the maximal horizontal distance of the inside of the rib cage), and cross-sectional area (CsA) (9), were measured at the T3–12 vertebral level, and the Haller index (HI) was calculated as the ratio of the TD of the chest to the AD of the chest (19, 20).

SAT thickness, including anterior subcutaneous adipose tissue (aSAT) thickness and posterior subcutaneous adipose tissue (pSAT) thickness, either in pre-arrest or post-arrest CT images, were measured at the T3–12 spinous process level.

aSAT thickness was measured as the distance from the sternum to the skin surface at the T3–T12 levels. pSAT thickness was measured as the distance from the posterior tip of the corresponding spinous process to the skin surface at the T3–T12 levels.

To minimize inter-rater measurement variability, 2 researchers underwent joint training and performed CT measurements according to a standardized operating procedure. Seven patients (10% of the CT Imaging Cohort) were independently assessed while blinded to clinical outcomes by both researchers. For each patient, measurements obtained at the T3–T12 vertebral levels were averaged separately according to CT time point, measurement type, and rater. Inter-rater reliability was evaluated using a two-way random-effects, absolute-agreement, single-measurement intraclass correlation coefficient [ICC (2, 1)] with 95% confidence intervals. The initial independent measurements obtained before repeat measurement or consensus review were used for the reliability analysis. Measurements differing by more than 5% were subsequently re-evaluated according to the standardized procedure, and disagreements were resolved by consensus. Detailed reliability estimates are provided in Supplementary file 1, Supplementary Table S1.

### Subgroup division

We divided all eligible patients into three subgroups given that positive mechanical ventilation could affect lung capacity (21): patients with or without positive mechanical ventilation were divided into different groups and analyzed. Specific ventilator parameters, including PEEP and tidal volume, were not matched because these data were not consistently available.

### Blood sampling and processing

Arterial blood sampling was initiated at the onset of CA, concurrently with CPR initiation and before ROSC or termination of resuscitation, irrespective of the subsequent resuscitation outcome. Sampling was performed irrespective of whether ROSC was subsequently achieved. Blood was drawn into EDTA-containing blood collection tubes. Samples were centrifuged at 2,000 × *g* for 10 min at 4 °C to separate plasma. The aliquots were immediately transferred to polypropylene microcentrifuge tubes and stored at −80 °C until further analysis to avoid repeated freeze-thaw cycles. Detailed pre-analytical procedures are provided in Supplementary file 1, Supplementary Table S2A.

Quantification of Adipokines Plasma concentrations of 12 adipokines were quantified using commercially available Enzyme-Linked Immunosorbent Assay (ELISA) kits according to the manufacturer's instructions. The 12 adipokines constituted a targeted candidate panel selected before outcome analysis on the basis of previous literature (22–25) concerning established and emerging adipose-derived mediators involved in metabolic regulation, systemic inflammation, endothelial function, and cardiovascular disorders. The panel included conventional adipokines and members of the CTRP family and was not derived from untargeted proteomic screening. The panel included: Adiponectin, Leptin, Resistin, Visfatin, Omentin-1, Vaspin, Chemerin, and the C1q/TNF-related protein family members (CTRP1, CTRP2, CTRP6, CTRP9, and CTRP12). Catalog numbers, assay ranges, analytical sensitivities, sample dilutions, and manufacturer-reported intra- and inter-assay CVs are provided in Supplementary file 1, Supplementary Table S2B. The laboratory technicians performing the ELISA assays were blinded to the patients' clinical data and neurological outcomes.

### Statistical analyses

Continuous data are expressed as the mean ± standard deviation or median and interquartile range, and categorical data are presented as counts with frequencies. Normally distributed continuous variables were compared by Student's *t-*test, while repeatedly measured thorax dimensions were compared with two-way ANOVA (for patients with the same type of ventilation mode while performing two CT scans), and skewed distributed continuous variables were compared by the Mann–Whitney *U*-test. Categorical variables were compared using the chi-square test.

For CT-derived measurements, the Benjamini–Hochberg false discovery rate (FDR) procedure was applied separately within each prespecified family of comparisons across the 10 vertebral levels from T3 to T12, according to measurement type, CT time point, outcome, and analysis population. Both unadjusted *P*-values and FDR-adjusted q values are reported.

To account for multiple comparisons across the 12 adipokines, *p*-values were adjusted using the Benjamini–Hochberg procedure in R to control the false discovery rate. Both unadjusted *p*-values and FDR-adjusted q values are reported. An FDR-adjusted *q*-value < 0.05 was considered statistically significant.

CPR duration and lactate were retained as clinical predictors for comparison with an extended model. To limit model complexity in this small cohort, one adipokine was selected after reviewing the FDR-adjusted group comparisons and the individual discriminatory performance of adipokines that met the FDR threshold. This selection was informed by the study data and was not prespecified. Logistic regression models were fitted to assess CPR duration alone, the clinical predictors together, and the clinical predictors with the selected adipokine added. All model comparisons used patients with complete data for the variables in the compared models. Odds ratios were reported per one-unit increase in each predictor.

Discrimination was summarized using the area under the receiver operating characteristic curve (AUC). DeLong 95% confidence intervals were calculated for apparent AUCs, and the clinical and extended models were compared using a paired DeLong test in the same patients. Calibration was assessed using a calibration plot, calibration slope, and Brier score. Internal validation used 1,000 bootstrap resamples to estimate optimism-corrected performance for each model with its predictors fixed. The data-informed adipokine selection step was not repeated within the bootstrap resamples. Firth logistic regression was used as a sensitivity analysis of the extended model.

No data were imputed. Analyses involving CTRP9 were performed using complete cases, whereas all 66 patients had complete data for neurological outcome, CPR duration, lactate, and CTRP6 and were included in the three prediction models.

A two-sided *p*-value < 0.05 was considered to indicate statistical significance. Analyses were conducted using SPSS statistical software version 24.0 (IBM Corporation, Armonk, NY) and GraphPad Prism 10.1.2 (GraphPad Software, San Diego, CA). The ROC curves were generated, and the 95% confidence intervals of the AUC were calculated using the DeLong method via the “pROC” package using R software version 4.6.1 (R Foundation for Statistical Computing, Vienna, Austria).

## Results

### Overview

A total of 1,663 patients who underwent CPR between May 31, 2019, and June 30, 2021, were identified in CT Imaging Cohort, of whom 60 patients also experienced OHCA prior to admission; 1,503 patients did not have the required CT images for longitudinal comparison. After screening, 100 patients met the inclusion criteria. Among them, 21 patients were excluded because of thoracotomy before either CT scan, and another five patients were excluded because of severe rib or sternum fracture due to thoracic trauma. Two patients had persistent pulmonary abnormalities that precluded comparable CT measurements. One patient experienced change in posture when post-arrest CT scans were performed (Supplementary file 2, Supplementary Figure S2), and one patient had congenital thoracic deformity. Thus, the final cohort included 70 patients (Figure 1).

**Figure 1:**
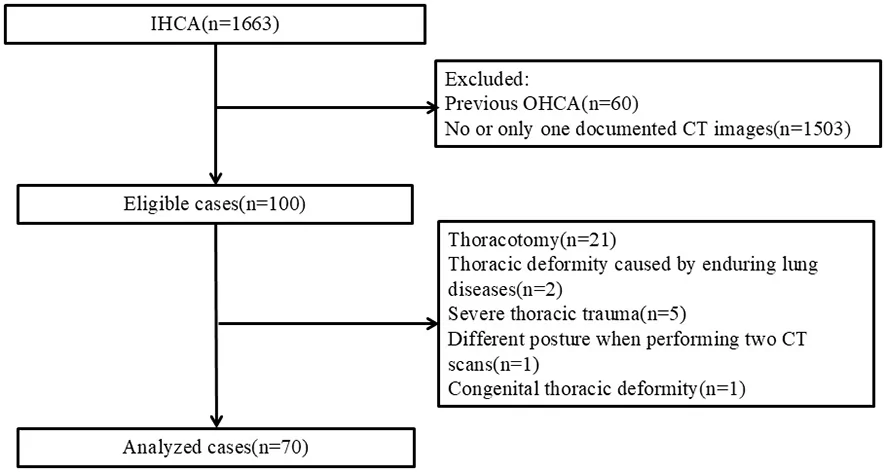
Flowchart of the study. IHCA, in-hospital cardiac arrest; OHCA, out-of-hospital cardiac arrest; CT, computed tomography.

A total of 206 patients who underwent cardiopulmonary resuscitation at the Emergency Department of West China Hospital between July 1, 2021, and February 28, 2022, were initially screened for inclusion in Biochemical Cohort. Among them, 36 patients were excluded due to the unavailability of blood specimens, 20 were excluded because of severe hemolysis that could interfere with the biochemical assays, leaving 150 patients for further eligibility assessment. Following the exclusion criteria, 2 patients aged under 18 years, 20 patients with OHCA, 11 patients with a neurological cause of cardiac arrest, 1 patient with traumatic cardiac arrest, 16 patients with hematological malignancies, 33 patients with end-stage chronic diseases;1 pregnant patient were excluded. Ultimately, 66 patients met all criteria and were enrolled in the final Biochemical Cohort for further analysis.

### Patient characteristics

Within each cohort, the baseline and arrest-related characteristics according to survival and neurological outcome are presented in Supplementary file 1, Supplementary Tables S3, S4. In the CT Imaging Cohort, pre-existing neurological impairment was more frequent among patients classified as having an unfavorable neurological outcome than among those classified as having a favorable neurological outcome before exclusion of these patients from the neurological outcome analysis [10/38 (26.3%) vs. 2/32 (6.3%), *P* = 0.031]. Therefore, the 12 patients with pre-existing neurological impairment were excluded from analyses of neurological outcome at hospital discharge, leaving 58 patients, including 30 with favorable and 28 with unfavorable neurological outcomes. For descriptive purposes, selected baseline and arrest-related characteristics of the CT Imaging Cohort (*n* = 70) and the Biochemical Cohort (*n* = 66) are presented in Table 1. The Biochemical Cohort had a lower rate of survival to hospital discharge than the CT Imaging Cohort [41/66 (62.1%) vs. 56/70 (80.0%), *P* = 0.021], whereas the proportion of patients classified as having a favorable neurological outcome did not differ significantly between the cohorts [24/66 (36.4%) vs. 32/70 (45.7%), *P* = 0.268]. No statistically significant differences were observed in the other characteristics presented in Table 1. Baseline characteristics of patients excluded because of severe hemolysis and those included in the Biochemical Cohort are presented in Supplementary file 1, Supplementary Table S5.

**Table 1:** Comparison of baseline characteristics between cohorts.

Variables	CT Imaging cohort (*n* = 70)	Biochemical cohort (*n* = 66)	*p*-value
Age (year; mean ± SD)	56.19 ± 17.47	60.71 ± 14.26	0.102
Male (%)	49 (70.0%)	48 (72.7%)	0.725
BMI	23.09 ± 4.43	22.78 ± 5.02	0.703
Comorbidities			
Heart disease (*n*, %)	31 (44.2%)	28 (42.4%)	0.826
Respiratory insufficiency (*n*, %)	15 (21.4%)	12 (18.2%)	0.635
Neurological insufficiency (*n*, %)	12 (17.1%)	11 (16.7%)	0.941
Gastrointestinal disease (*n*, %)	7 (10.0%)	4(6.1%)	0.400
Renal insufficiency (*n*, %)	6 (8.6%)	10 (15.2%)	0.234
Sepsis (*n*, %)	11 (15.7%)	11 (16.7%)	0.880
Malignancy (*n*, %)	5 (7.1%)	10 (15.2%)	0.171
CA characteristics			
Initial rhythm-shockable (*n*, %)	15 (21.4%)	17 (25.8%)	0.552
CPR duration, min	6 (3,13)	9 (4,13)	0.080
Outcomes			
Survival to hospital discharge (*n*, %)	56 (80.0%)	41 (62.1%)	0.021^*^
Favorable neurological outcome (*n*, %)	32(45.7%)	24(36.4%)	0.268

Data are presented as mean ± SD, median (IQR), or n (%) as appropriate. Significance annotations reflect P-values: ^*^P < 0.05.

### Associations between CT-derived indices and patient outcomes

#### Cardiac arrest characteristics

The median number of cardiac arrests during hospitalization was one across all patients, and the median duration of CPR was 6 min. Overall, 65 of 70 patients (92.9%) underwent tracheal intubation. The proportions of patients intubated before cardiac arrest or during CPR did not differ significantly between survivors and non-survivors (Supplementary file 1, Supplementary Tables S6, S7).

Pre-arrest intubation was less frequent among patients with favorable neurological outcomes than among those with unfavorable neurological outcomes [11/30 (36.7%) vs. 21/28 (75.0%), *P* = 0.003], whereas the proportion intubated during CPR did not differ significantly between the groups [14/30 (46.7%) vs. 16/28 (57.1%), *P* = 0.425] (Supplementary file 1, Supplementary Tables S8, S9).

#### Timing of CT examinations and intervening cardiac arrest characteristics

The median time interval between the recorded CT and the first CA of all patients was 2.84 days, whereas the interval between the ROSC and the first rechecked CT was 6.35 days. Neither interval differed significantly between survivors and non-survivors. The interval from ROSC to the post-arrest CT examination was numerically longer in the favorable-outcome group than in the unfavorable-outcome group (9.69 vs. 5.61 days, *P* = 0.056), although the difference did not reach statistical significance (Supplementary file 1, Supplementary Tables S10, S11).

The number of cardiac arrest events and cumulative CPR duration between the two CT examinations did not significantly differ among patients with different outcomes during the CT interval (Supplementary file 1, Supplementary Tables S12, S13).

#### CT-derived indices and patient outcomes

Inter-rater reliability was high for adipose tissue thickness, anteroposterior diameter, and cross-sectional area, whereas the estimates for transverse diameter were less precise, with wider confidence intervals (Supplementary file 1, Supplementary Table S1).

#### Bony thorax measurements and outcomes

No significant differences in TD, AD, CsA, or HI at different vertebral levels were found between patients with different outcomes on pre-arrest or post-arrest CT images. No statistically significant differences were observed in the prespecified ventilation-related subgroup analyses (Supplementary file 1, Supplementary Tables S16–S19, S22–S25, S28–S31, S34–S37).

#### SAT measurements and outcomes

Neither aSAT measurements nor pSAT thickness differed significantly between survivors and non-survivors in the overall CT Imaging Cohort or in the ventilation-related subgroup analyses (Supplementary file 1, Supplementary Tables S14, S15, S20, S21, S26, S27, S32, S33).

After excluding 12 patients with pre-existing neurological impairment, post-arrest pSAT thickness was greater at several spinous process levels in patients with favorable neurological outcomes in the unadjusted analyses; however, none of the between-group differences remained statistically significant after BH-FDR correction (all *q* ≥ 0.125; Table 2). Pre-arrest pSAT thickness was found no significant difference in the unadjusted analyses. A sensitivity analysis was subsequently restricted to patients who had the same ventilation status during both CT examinations to reduce potential confounding by ventilation. In the ventilation-matched sensitivity analysis, patients with favorable neurological outcomes had greater pre-arrest pSAT thickness at several spinous process levels. After BH-FDR correction across T3–T12, the between-group differences remained significant at T8–T11 (Supplementary file 1, Supplementary Table S27); pSAT thickness was significantly thicker on post-arrest CT images at the T8–12 spinous process level in patients with indicators of favorable neurological prognosis than in patients with indicators of poor neurological prognosis, with these differences remaining significant after BH-FDR correction (Supplementary file 1, Supplementary Table S33).

**Table 2:** Posterior SAT thickness at each spinous process level measured among patients with different outcomes at discharge on post-arrest CT images.

pSAT thickness (mm) (mean ±SD)	Favorable neurological outcome (*n* = 30)	Unfavorable neurological outcome (*n* = 28)	*P*-value	BH *q*-value
T3	22.31 ± 10.89	19.98 ± 9.10	0.404	0.404
T4	20.70 ± 9.53	18.63 ± 7.89	0.383	0.404
T5	20.45 ± 8.26	17.05 ± 7.09	0.103	0.147
T6	19.48 ± 9.08	15.95 ± 5.86	0.086	0.143
T7	18.94 ± 9.03	14.97 ± 5.52	0.050	0.125
T8	18.65 ± 9.10	14.40 ± 5.76	0.038	0.125
T9	19.20 ± 9.67	15.07 ± 6.45	0.063	0.126
T10	19.19 ± 9.64	14.40 ± 6.96	0.035	0.125
T11	19.19 ± 9.98	14.33 ± 7.54	0.047	0.125
T12	17.58 ± 9.58	14.80 ± 8.52	0.309	0.386

P-values are unadjusted. BH q-values were calculated using the Benjamini–Hochberg procedure across the 10 vertebral-level comparisons from T3 to T12. Statistical significance after multiplicity correction was defined as q < 0.05.

pSAT, posterior subcutaneous adipose tissue; SD, standard deviation.

Although the CT-derived pSAT findings were attenuated after correction for multiple testing, the observed anatomical patterns prompted us to explore whether circulating adipose-related factors were associated with neurological outcomes. We therefore evaluated adipokine profiles measured at the time of cardiac arrest in the independent Biochemical Cohort.

### Association between circulating adipokines and patient outcomes

#### Plasma adipokine profiles

In the Biochemical Cohort, plasma concentrations of 12 adipokines measured at the time of cardiac arrest were compared between 24 patients with favorable and 42 patients with unfavorable neurological outcomes. After Benjamini–Hochberg correction, CTRP6 (*q* < 0.001), CTRP9 (*q* = 0.031), Omentin-1 (*q* = 0.019), and Vaspin (*q* = 0.031) were higher in the favorable-outcome group, whereas Chemerin was lower (*q* = 0.008). The differences in Adiponectin, CTRP12, Resistin, CTRP1, CTRP2, Leptin, and Visfatin did not remain statistically significant after FDR correction (Table 3 and Figures 2A–L). These comparisons were considered exploratory.

**Table 3:** Comparison of plasma adipokine levels between neurological outcome groups in biochemical cohort.

Adipokine	Unfavorable Outcome (*n* = 42)	Favorable outcome (*n* = 24)	*P*-value	FDR-adjusted *q*-value
Higher concentrations in the favorable-outcome group after FDR correction				
CTRP6 (ng/mL)	3.13 (2.71–3.63)	4.01 (3.28–4.52)	< 0.001^*^	< 0.001^*^
CTRP9 (ng/mL)	127.78 (90.66–189.31)	192.80 (150.98–269.03)	0.013^*^	0.031^*^
Omentin-1 (ng/mL)	37.59 ± 11.45	46.64 ± 13.12	0.005^*^	0.019^*^
Vaspin (ng/mL)	7.43 (4.42–9.50)	8.98 (7.50–11.51)	0.011^*^	0.031^*^
Lower concentrations in the favorable-outcome group after FDR correction				
Chemerin (ng/mL)	623.87 ± 155.13	493.22 ± 147.85	0.001^*^	0.008^*^
No FDR-supported between-group difference				
Adiponectin (μg/mL)	22.93 (17.14–27.60)	24.08 (20.60–36.44)	0.056	0.084
CTRP1 (ng/mL)	647.95 (486.62–804.26)	588.03 (469.93–733.10)	0.266	0.290
CTRP2 (ng/mL)	19.50 (14.90–22.28)	21.95 (16.27–24.55)	0.144	0.173
CTRP12 (ng/mL)	5.35 (3.80–6.54)	5.53 (4.77–7.68)	0.036^*^	0.073
Leptin (ng/mL)	16.65 ± 3.30	15.18 ± 3.34	0.054	0.084
Visfatin (ng/mL)	80.08 (61.78–105.18)	81.70 (59.49–95.73)	0.826	0.826
Resistin (ng/mL)	49.05 ± 10.33	43.91 ± 10.03	0.054	0.084

Data are presented as mean ± standard deviation or median (interquartile range), as appropriate. Between-group comparisons were performed using Student's t-test or the Mann–Whitney U-test. FDR-adjusted q-values were calculated across the 12 adipokine comparisons using the Benjamini–Hochberg procedure. CTRP, C1q/TNF-related protein; FDR, false discovery rate. Significance annotations reflect P-values and FDR-adjusted q-values: ^*^P < 0.05, ^*^q < 0.05.

**Figure 2:**
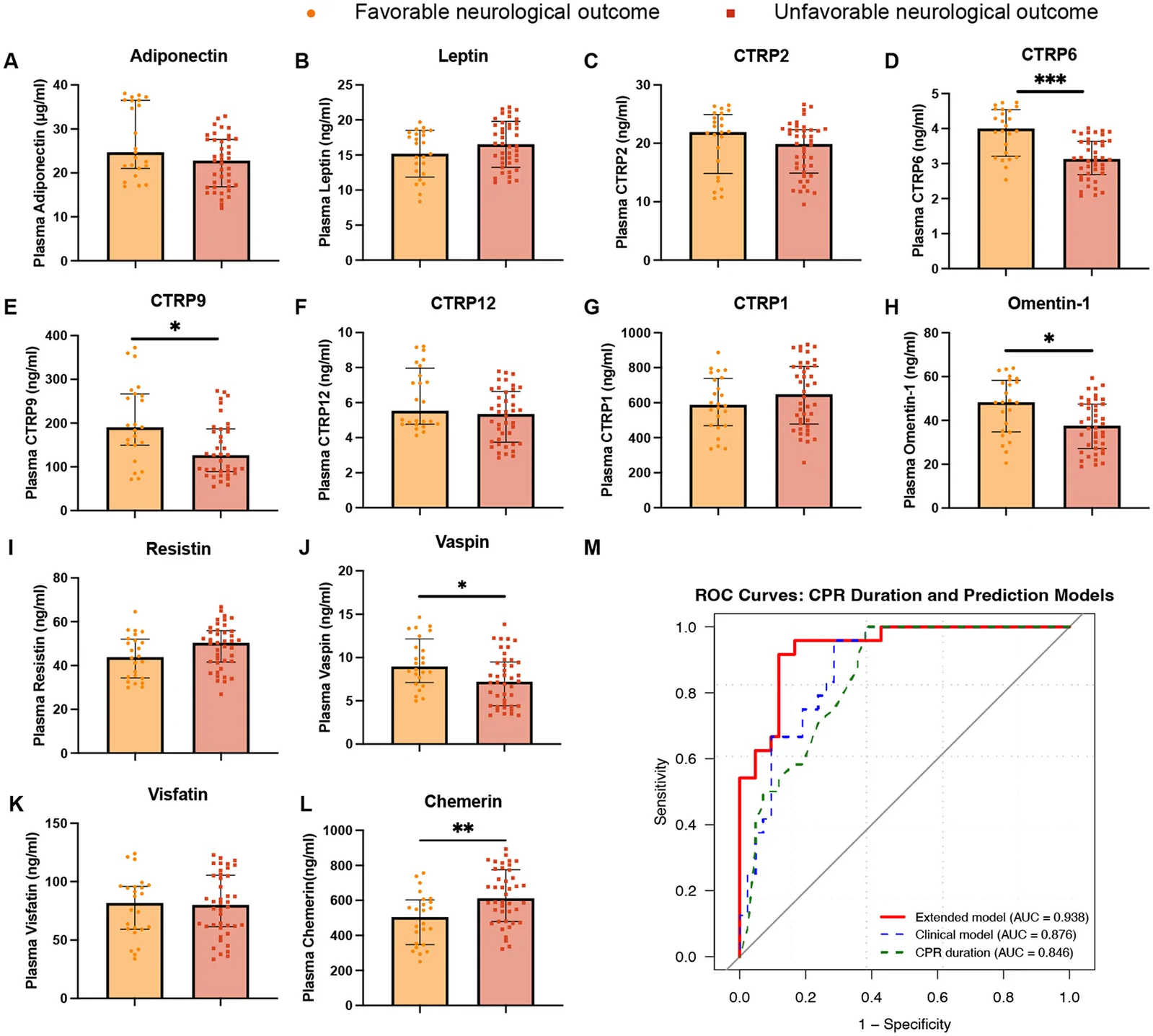
Circulating adipokine concentrations and exploratory model discrimination in Biochemical Cohort. **(A–L)** Circulating concentrations of the 12 adipokines according to neurological outcome at hospital discharge. Data are presented as mean with standard deviation or median with interquartile range, as appropriate. Between-group *p*-values were calculated using Student's *t*-test or the Mann–Whitney *U*-test. Significance annotations reflect FDR-adjusted *q-*values: *q < 0.05, **q < 0.01, and ***q < 0.001. **(M)** Receiver operating characteristic curves for CPR duration alone, the clinical model containing CPR duration and lactate, and the extended model adding CTRP6. The apparent AUCs were 0.846 (95% CI, 0.755–0.937), 0.876 (95% CI, 0.795–0.957), and 0.938 (95% CI, 0.882–0.993), respectively. The apparent AUC difference between the extended and clinical models was 0.062 (95% CI, −0.003 to 0.126; paired DeLong *p* = 0.062). AUC, area under the receiver operating characteristic curve; CTRP, C1q/TNF-related protein; FDR, false discovery rate.

#### Exploratory prediction models in biochemical cohort

Among the five adipokines meeting the FDR threshold, CTRP6 had the highest apparent individual AUC of 0.812 (95% CI, 0.698–0.926) and was selected in a data-informed manner for inclusion in the exploratory extended model. The individual AUCs of the five FDR-supported adipokines are presented in Supplementary file 1, Supplementary Table S38.

All three prediction models were evaluated in the same 66 patients, including 24 patients with a favorable neurological outcome. In the clinical model, longer CPR duration (OR per minute, 0.877; 95% CI, 0.813–0.946; *P* < 0.001) and higher lactate concentration (OR per mmol/L, 0.816; 95% CI, 0.669–0.996; *P* = 0.045) were associated with lower odds of a favorable neurological outcome. In the exploratory extended model, longer CPR duration remained associated with lower odds of a favorable neurological outcome (OR per minute, 0.862; 95% CI, 0.776–0.958; *P* = 0.006), whereas higher CTRP6 concentration was associated with higher odds of a favorable neurological outcome (OR per ng/mL, 8.880; 95% CI, 2.179–36.187; *P* = 0.002). Lactate was not statistically significant in the extended model (OR per mmol/L, 0.852; 95% CI, 0.669–1.086; *P* = 0.196; Table 4A). Complete model coefficients are reported in Supplementary file 1, Supplementary Table S39.

**Table 4:** Exploratory prediction models for favorable neurological outcome in the biochemical cohort.

(A) Logistic regression coefficients				
Model and predictor		OR (95% CI)		*P*-value
Clinical model				
CPR duration, per 1 min		0.877 (0.813–0.946)		< 0.001
Lactate, per 1 mmol/L		0.816 (0.669–0.996)		0.045
Extended model				
CPR duration, per 1 min		0.862 (0.776–0.958)		0.006
Lactate, per 1 mmol/L		0.852 (0.669–1.086)		0.196
CTRP6, per 1 ng/mL		8.880 (2.179–36.187)		0.002
**(B) Model performance and internal validation**				
**Model**	**Apparent AUC (95% CI)**	**Optimism-corrected AUC**	**Optimism-corrected calibration slope**	**Optimism-corrected Brier score**
CPR duration	0.846 (0.755–0.937)	0.843	0.979	0.165
Clinical model: CPR duration + lactate	0.876 (0.795–0.957)	0.868	0.941	0.152
Extended model: CPR duration + lactate + CTRP6	0.938 (0.882–0.993)	0.930	0.885	0.115

Paired comparison of the extended vs. clinical model: apparent AUC difference, 0.062 (95% CI, −0.003 to 0.126); DeLong P = 0.062.

Odds ratios were estimated using ordinary logistic regression and are expressed per unit increase as indicated. AUC confidence intervals and the paired AUC difference were calculated using the DeLong method. Optimism-corrected estimates were obtained from 1,000 bootstrap resamples of models with fixed predictors. CTRP6 was selected after examination of the cohort data; this selection step was not repeated within bootstrap resamples. AUC, area under the receiver operating characteristic curve; CI, confidence interval; CTRP, C1q/TNF-related protein.

In the Firth logistic regression sensitivity analysis, the direction of the associations was unchanged. CPR duration remained inversely associated with favorable neurological outcome (OR per minute, 0.882; 95% CI, 0.787–0.954; *P* < 0.001), and CTRP6 remained positively associated with favorable neurological outcome (OR per ng/mL, 6.433; 95% CI, 2.120–27.636; *P* < 0.001), whereas lactate was not statistically significant (OR per mmol/L, 0.880; 95% CI, 0.681–1.067; *p* = 0.211; Supplementary file 1, Supplementary Table S40).

The apparent AUCs were 0.846 (95% CI, 0.755–0.937) for CPR duration alone, 0.876 (95% CI, 0.795–0.957) for the clinical model, and 0.938 (95% CI, 0.882–0.993) for the extended model (Figure 2M). Although the extended model had a numerically higher apparent AUC than the clinical model, the difference did not reach statistical significance (ΔAUC, 0.062; 95% CI, −0.003 to 0.126; paired DeLong *P* = 0.062; Table 4B).

After 1,000 bootstrap resamples, the optimism-corrected AUCs were 0.843, 0.868, and 0.930 for the CPR-duration, clinical, and extended models, respectively. The optimism-corrected calibration slope of the extended model was 0.885, and its optimism-corrected Brier score was 0.115. Full bootstrap-corrected performance estimates are reported in Supplementary file 1, Supplementary Table S41, and the bootstrap-corrected calibration plots for the clinical and extended models are shown in Supplementary file 2, Supplementary Figure S3.

## Discussion

In this study, we conducted two parallel exploratory analyses in independent cohorts of patients with IHCA. In the overall CT Imaging Cohort, unadjusted differences in post-arrest pSAT thickness did not remain significant after FDR correction, whereas selected differences were observed in the exploratory ventilation-matched sensitivity analysis. In the biochemical cohort, several circulating adipokines differed between neurological outcome groups after correction for multiple comparisons. Because CT-derived pSAT thickness and circulating adipokines were not measured in the same patients, these findings and should not be interpreted as evidence of an integrated multimodal model.

Unlike previous studies (9, 26–28) that evaluated broad, unselected CA populations, our study specifically targets the factors affecting neurological prognosis exclusively in CA survivors. Consequently, all patients included in CT Imaging Cohort successfully achieved ROSC. In this strictly defined subgroup, 80% survived to discharge, with 45.7% achieving favorable neurological outcomes. Given the paucity of data regarding the anatomical thoracic characteristics of ROSC patients receiving advanced life support (ALS) in the intensive care unit (ICU), our selected cohort accurately represents the specific sub-population that survives the initial ischemic insult. Investigating this selected group is clinically imperative, as it provides critical insights for ICU practitioners who frequently face the challenging dilemma of early neurological prognostication and the potential WLST in unconscious post-arrest patients. Accordingly, the extended model should be regarded as hypothesis-generating and requires external validation before its potential use alongside existing prognostic tools can be considered.

The CT Imaging Cohort showed exploratory differences in pSAT thickness according to neurological outcome at selected thoracic levels; however, these findings do not establish a causal relationship, and their biological basis remains uncertain. Traditionally viewed merely as an energy storage depot, pSAT is increasingly recognized as a highly active endocrine organ. In the acute phase following IHCA and ROSC, the systemic ischemia-reperfusion cascade triggers a massive stress response. In this context, adipose tissue has endocrine functions and releases bioactive mediators; however, the present study did not determine the tissue source of the circulating adipokines or establish a relationship between pSAT thickness and adipokine concentrations. To determine whether this survival advantage stems from the metabolic activity of the adipose tissue rather than mere anatomical insulation, we analyzed the secretory profile of these patients in the independent Biochemical Cohort. The biochemical findings from Biochemical Cohort provide a separate observation that circulating adipokine profiles may differ according to neurological outcome after IHCA. However, because adipokine concentrations and pSAT thickness were not assessed in the same patients, the present data cannot determine whether the anatomical findings in the CT Imaging Cohort are related to the circulating adipokine patterns observed in the Biochemical Cohort. Patients with favorable neurological outcomes had higher circulating concentrations of CTRP6, CTRP9, Omentin-1, and Vaspin and a lower concentration of Chemerin after FDR correction. The numerical increase in AUC after adding CTRP6 suggests potential incremental prognostic information beyond CPR duration and lactate. However, the paired AUC difference was not statistically significant, and the small single-center sample limits the precision and generalizability of the estimates. CTRP6 should therefore be regarded as a candidate biomarker for further investigation rather than a clinically applicable prognostic tool at present. These parallel observations generate a hypothesis regarding possible relationships among adipose distribution, circulating adipokines, and neurological recovery (Figure 3), which requires direct evaluation in patients undergoing concurrent imaging and biomarker measurement.

**Figure 3:**
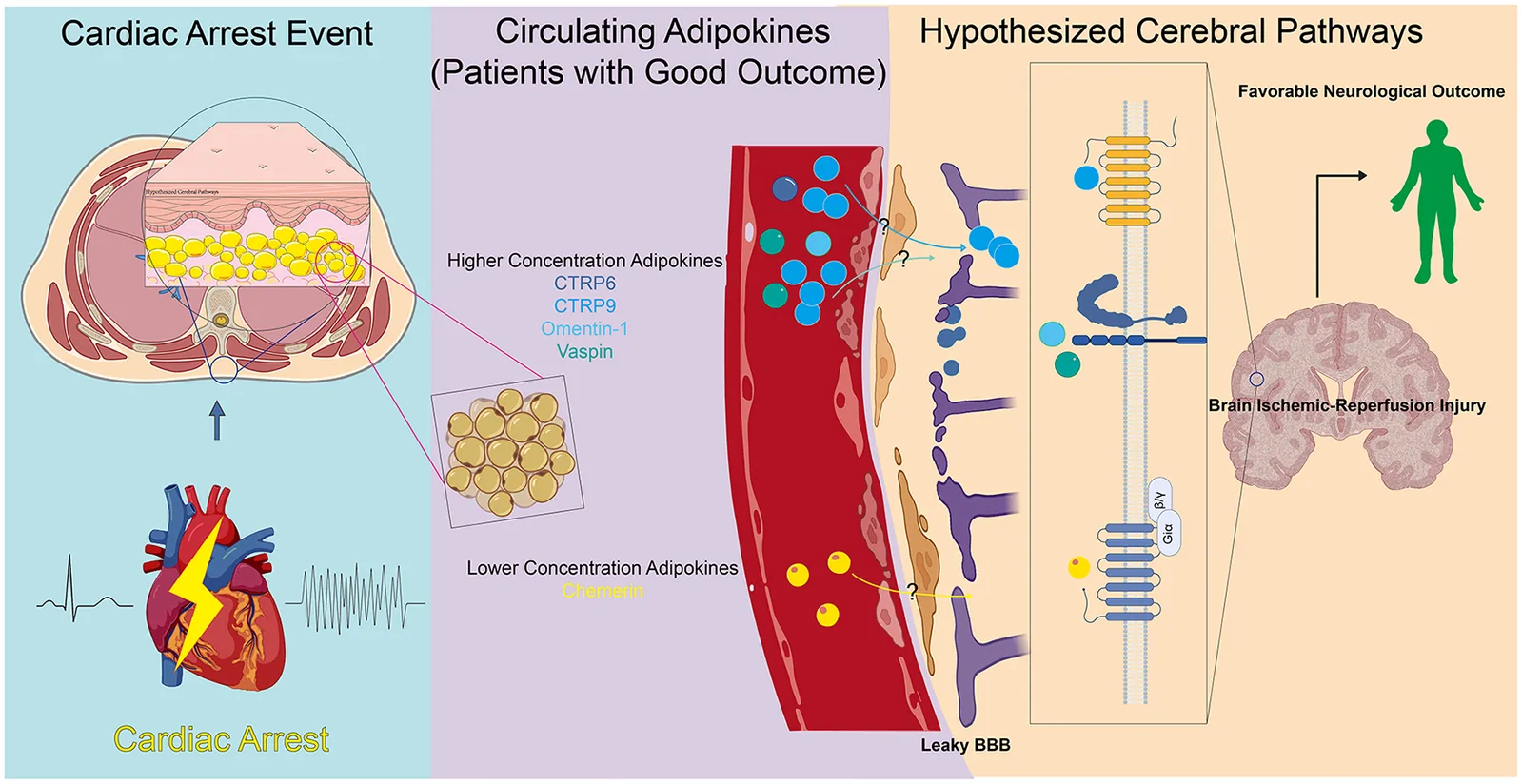
Hypothetical framework relating thoracic subcutaneous adipose tissue, circulating adipokines, and neurological outcome after in-hospital cardiac arrest. In CT Imaging Cohort, post-arrest posterior subcutaneous adipose tissue (pSAT) thickness was examined in relation to neurological outcome. In the Biochemical Cohort, blood samples were obtained at the time of cardiac arrest, irrespective of subsequent return of spontaneous circulation. Compared with patients who had an unfavorable neurological outcome at hospital discharge, those with a favorable outcome had higher circulating concentrations of CTRP6, CTRP9, Omentin-1, and Vaspin and a lower concentration of Chemerin; these differences remained significant after false discovery rate correction. The proposed links between pSAT and circulating adipokines, adipokine passage across the blood–brain barrier, receptor binding, and effects on neurological injury are hypothetical and were not assessed in this study. pSAT and adipokines were measured in different patients, and their relationship did not evaluate within individuals. CA, cardiac arrest; CTRP, C1q/TNF-related protein; BBB, Blood-Brain Barrier.

Chemerin showed a direction of association opposite to that of CTRP6, CTRP9, Omentin-1, and Vaspin. Although Chemerin has been implicated in inflammatory and metabolic pathways, its biological effects appear to be context-dependent, and both pro-inflammatory and potentially protective effects have been reported (29–31). The present study measured circulating Chemerin at a single time point and did not assess its tissue source, receptor signaling, or effects within the central nervous system. Therefore, the observed association should not be interpreted as evidence that reduced Chemerin is neuroprotective or that Chemerin contributes causally to post-arrest brain injury.

The “obesity paradox” in CA patients suggests that moderately overweight patients often exhibit better survival and neurological outcomes compared to underweight or severely obese individuals (11–13). However, conflicting data exist regarding metrics like thoracic diameter (27), and the underlying protective mechanisms remain poorly defined. Although linear BMI-SAT correlations exist in specific populations (e.g., morbidly obese individuals or healthy screenings) (10), our study revealed no significant BMI differences between any prognostic groups. Because most participants had BMI values within the normal range, these findings cannot be directly generalized to overweight or obese populations. The results suggest only that regional pSAT thickness may capture anatomical information not fully represented by BMI. Consequently, our findings suggest that regional pSAT may provide information not captured by BMI, although causality cannot be inferred. These findings align with previous reports linking deeper pSAT to better short-term survival via enhanced compression efficacy, further extending this anatomical advantage to favorable neurological recovery in CA survivors.

The reason why outcome-related differences were observed for pSAT but not aSAT remains uncertain. Anterior and posterior thoracic SAT differ in anatomical location, baseline thickness, and potential exposure to mechanical forces during chest compression and supine positioning. Previous IHCA studies have reported inconsistent associations of anterior and posterior SAT with clinical outcomes, suggesting that these findings may depend on the study population, measurement site, and outcome definition (9, 10, 32). Because the pSAT differences in the overall analysis did not remain significant after FDR correction, a depot-specific biological effect cannot be inferred, and measurement variability, residual confounding, or chance remain possible explanations.

Patients with smaller thoracic cages more easily acquire an adequate compression depth and thoracic cage compression ratio, leading to greater intrathoracic pressure and better systemic blood perfusion under the same chest compression maneuver (9). We did not find any studies comparing chest dimensions twice during the pericardiac arrest period. In addition, whether the thorax will deform after CPR has not been studied. Our work did not reveal any difference in bony thorax dimensions (TD, AD, or CsA) or shape (HI) between survivors and non-survivors or between patients with different degrees of neurological recovery.

Several confounding factors could affect the thoracic cage dimension measurements. First, the anatomical structure of the fourth intercostal plane varies among different individuals (Supplementary file 2, Supplementary Figure S4) (33). Second, positive mechanically ventilated patients were unable to synchronize with CT scanning instructions; thus, the images were randomly captured during either inspiration or expiration. Third, the level of positive end-expiratory pressure (PEEP) can influence the end-expiratory lung volume (EELV) (21). Accordingly, we divided patients based on the type of respiratory support received during the two chest scans, and we innovatively measured the whole thorax from different spinous processes and vertebral levels to offset the effects of individual anatomical variation and mechanical ventilation. Surprisingly, CA patients with favorable neurological outcomes across different subgroups had thicker pSAT thickness at lower spinous process levels, both before and after chest compression.

While the parallel cohort observations generate a hypothesis regarding a potential “adipose-brain axis” mediated by specific adipokines, the precise causal mechanisms and molecular signaling pathways require further experimental validation. Future studies combining concurrent imaging, serial biomarker sampling, and experimental models are needed to evaluate the proposed biological pathways.

## Limitations

Several limitations warrant consideration. First, the retrospective design and strict requirement for serial CT scans inherently introduce substantial selection bias. Without comparative validation against the eligible-but-excluded cohort, the magnitude of this bias remains unquantified, and the external validity of our findings is uncertain. Consequently, our findings may reflect a cohort with longer hospital stays or different baseline prognoses compared to the general unselected ICU post-arrest population, warranting cautious interpretation regarding the broad generalizability of these anatomical findings. Furthermore, our modest sample size yields a low events-per-variable (EPV) ratio of eight, requiring cautious interpretation of the multivariable model due to potential statistical overfitting. Second, the large number of comparisons across vertebral levels and subgroups increased the risk of false-positive findings. Although FDR adjustment was applied within defined comparison families, the CT findings—particularly those from small sensitivity subgroups—remain exploratory. Third, the observational nature precludes causal inference; whether these adipokine shifts are actively neuroprotective or merely proxy indicators of baseline metabolic reserves necessitates mechanistic validation using targeted *in vivo* models. Fourth, because Our two cohorts were temporally non-overlapping, the link between structural pSAT thickness and the functional adipokine profile remains inferential. Furthermore, the exclusion of patients with cachexia, end-stage chronic disease, and hematological malignancy from the Biochemical Cohort introduces potential population heterogeneity. Prospective studies featuring simultaneous CT imaging and blood sampling are strictly required to validate this within-patient correlation. Finally, our assessment was restricted to pSAT thickness based on thoracic CT imaging which could be confounded by intubation, unmeasured ventilation parameters, CT timing, fluid status, and biomarkers were quantified at a single time point. Visceral adipose tissue that secreting adipokines were not evaluated in our study. Thus, SAT thickness does not fully represent adipose tissue distribution or endocrine activity. Future initiatives must explore the contributions of visceral adiposity and incorporate serial sampling to map post-resuscitation temporal dynamics.

## Conclusion

In conclusion, the CT Imaging Cohort and Biochemical Cohort provided independent exploratory assessments of anatomical and circulating adipose-related markers in relation to outcomes after IHCA. In the CT Imaging Cohort, associations between pSAT thickness and neurological outcome were attenuated after correction for multiple testing, whereas several adipokines measured at the time of cardiac arrest, including CTRP6, CTRP9, and Chemerin, showed outcome-related differences in the Biochemical Cohort. These findings support further investigation of circulating adipokines as candidate markers of neurological outcome and of the hypothesized adipose–brain axis after cardiac arrest. Addition of CTRP6 to CPR duration and lactate numerically improved model discrimination, although the incremental difference in AUC did not reach statistical significance and the model remains susceptible to overfitting because of the limited sample size. External validation in larger cohorts is required before its prognostic or clinical utility can be determined. The two independent exploratory analyses provide complementary anatomical and biochemical observations but do not establish a direct mechanistic link between CT-derived pSAT thickness and circulating adipokines. Accordingly, the present findings should not be interpreted as evidence of an integrated multimodal prediction model or a causal adipose–brain pathway.

### Transparency, rigor, and reproducibility statement

To ensure the rigor and reproducibility of this study, strict methodological standards were applied throughout the study design, data collection, and analysis. Pre-established inclusion and exclusion criteria were strictly adhered to for patient selection in both the CT Imaging Cohort and the Biochemical Cohort to minimize selection bias. The sample sizes were primarily determined based on the availability of eligible clinical records and high-quality imaging/blood samples during the specified study period. For the biochemical analysis in Biochemical Cohort, all enzyme-linked immunosorbent assays (ELISA) were performed by laboratory personnel who were strictly blinded to the patients' clinical data and neurological outcomes. The primary data collection procedures and outcome definitions were established before the present reanalysis. The FDR-adjusted comparisons and revised exploratory prediction models were conducted in response to peer-review concerns and are described transparently in the Methods. Detailed methodologies, including specific commercially available ELISA kits and CT measurement parameters, are thoroughly documented in the Methods section to allow for independent replication. De-identified raw data supporting the conclusions of this study are available from the corresponding author upon reasonable request.

## Data Availability

The raw data supporting the conclusions of this article will be made available by the authors, without undue reservation.
