# Supplementary material for: CT-derived subcutaneous adipose tissue thickness and circulating adipokines in relation to neurological outcome after in-hospital cardiac arrest: two parallel cohort analyses
Source: Front Neurol. 2026 Oct 20;17:1898760. doi: 10.3389/fneur.2026.1898760 (PMC13727567; doi:10.3389/fneur.2026.1898760)
Supplement: Supplementary file 1 [file Table_1.docx]

**Supplementary file 1**

**Table S1. Inter-rater reliability of CT-derived measurements in seven patients**

| **Measurement** | **CT time point** | **Patients, n** | **Rater 1 mean ± SD** | **Rater 2 mean ± SD** | **Mean difference** | **ICC (95% CI)** |
| --- | --- | --- | --- | --- | --- | --- |
| aSAT thickness, mm | Pre-arrest | 7 | 14.42 ± 6.42 | 14.32 ± 6.33 | 0.09 | 0.999 (0.996–1.000) |
| aSAT thickness, mm | Post-arrest | 7 | 13.91 ± 7.96 | 13.88 ± 8.05 | 0.02 | 1.000 (0.998–1.000) |
| pSAT thickness, mm | Pre-arrest | 7 | 19.36 ± 5.95 | 19.23 ± 5.86 | 0.13 | 0.999 (0.994–1.000) |
| pSAT thickness, mm | Post-arrest | 7 | 18.86 ± 5.05 | 18.85 ± 5.41 | 0.01 | 0.997 (0.980–0.999) |
| Transverse diameter, mm | Pre-arrest | 7 | 230.31 ± 10.90 | 231.77 ± 10.73 | -1.46 | 0.880 (0.486–0.978) |
| Transverse diameter, mm | Post-arrest | 7 | 233.76 ± 9.90 | 232.53 ± 10.94 | 1.23 | 0.835 (0.318–0.970) |
| Anteroposterior diameter, mm | Pre-arrest | 7 | 108.28 ± 18.32 | 108.58 ± 18.32 | -0.30 | 0.995 (0.975–0.999) |
| Anteroposterior diameter, mm | Post-arrest | 7 | 108.15 ± 16.65 | 107.24 ± 16.71 | 0.92 | 0.995 (0.969–0.999) |
| Cross-sectional area, cm² | Pre-arrest | 7 | 289.47 ± 30.49 | 291.60 ± 32.12 | -2.12 | 0.977 (0.885–0.996) |
| Cross-sectional area, cm² | Post-arrest | 7 | 296.39 ± 28.54 | 296.45 ± 24.71 | -0.06 | 0.985 (0.916–0.997) |
| Note: For each patient, measurements across T3–T12 were averaged separately by CT time point, measurement type, and rater. Inter-rater reliability was estimated using a two-way random-effects, absolute-agreement, single-measurement intraclass correlation coefficient [ICC(2,1)] with 95% confidence intervals. Mean difference was calculated as Rater 1 minus Rater 2. Cross-sectional area values exported in mm² were divided by 100 for presentation in cm². HI was not included because it was derived from the measured thoracic diameters rather than independently measured. pSAT, posterior subcutaneous adipose tissue; aSAT, anterior subcutaneous adipose tissue; ICC, intraclass correlation coefficient; SD, standard deviation. | | | | | | |
|  |  |  |  |  |  |  |
|  |  |  |  |  |  |  |

**Table S2. Pre-analytical procedures and technical characteristics of the ELISA assays used for adipokine measurements in the Biochemical Cohort**

**A. Sample collection and pre-analytical procedures**

| **Item** | **Information** |
| --- | --- |
| Study cohort | Biochemical Cohort |
| Sampling time | During cardiac arrest and ongoing resuscitation, before ROSC or termination of resuscitation. |
| Relationship to ROSC | Sampling was performed irrespective of whether ROSC was subsequently achieved |
| Blood source | Arterial blood |
| Collection tube | EDTA anticoagulant tube |
| Sample matrix | EDTA plasma |
| Time from collection to centrifugation | Less than 12h |
| Centrifugation conditions | 2,000 × *g* for 10 min at 4°C |
| Aliquoting | Plasma was divided into 200-μL aliquots in polypropylene microcentrifuge tubes. |
| Storage conditions | Stored at −80°C until analysis; maximum storage duration: 6 months |
| Freeze-thaw cycles | No more than two freeze–thaw cycles |
| Hemolysis assessment | Samples with severe hemolysis were excluded before ELISA measurement |
| Samples excluded for severe hemolysis | 20 |
| Assay replicates | Each sample was measured once. |
| Plate allocation and batch handling | Samples were randomly allocated to assay plates, with favorable- and unfavorable-outcome samples distributed across plates. |
| Blinding | Laboratory personnel were blinded to neurological outcomes |

**B. Technical characteristics of the ELISA assays**

| **Adipokine** | **Manufacturer** | **Catalogue number** | **Sample matrix** | **Reported unit** | **Assay range** | **Manufacturer-reported analytical** | **Sample dilution** | **Intra-assay CV** | **Inter-assay CV** |
| --- | --- | --- | --- | --- | --- | --- | --- | --- | --- |
| Adiponectin | Quanzhou Ruixin Biotechnology Co., Ltd | RX104694H | EDTA plasma | μg/mL | 0.9375μg/mL-30μg/mL | <0.1 μg/mL | 1:5 | <10% | <15% |
| Leptin | Quanzhou Ruixin Biotechnology Co., Ltd | RX105238H | Plasma | ng/mL | 0.5ng/mL-16ng/mL | <0.1 ng/mL | 1:5 | <10% | <15% |
| CTRP1 | Quanzhou Ruixin Biotechnology Co., Ltd | RX106408H | Plasma | ng/mL | 25ng/mL-800ng/mL | <1 ng/mL | 1:5 | <10% | <15% |
| Resistin | Quanzhou Ruixin Biotechnology Co., Ltd | RX105994H | Plasma | ng/mL | 1.5ng/mL-48ng/mL | <0.1 ng/mL | 1:5 | <10% | <15% |
| Chemerin | Quanzhou Ruixin Biotechnology Co., Ltd | RX103386H | Plasma | ng/mL | 25ng/ml-800ng/ml | <1 ng/mL | 1:5 | <10% | <15% |
| Omentin-1 | Quanzhou Ruixin Biotechnology Co., Ltd | RX105141H | Plasma | ng/mL | 2ng/mL-64ng/mL | <0.1 ng/mL | 1:5 | <10% | <15% |
| CTRP12 | Quanzhou Ruixin Biotechnology Co., Ltd | RX103295H | Plasma | ng/mL | 0.25ng/mL-8ng/mL | <0.1 ng/mL | 1:5 | <10% | <15% |
| CTRP6 | Quanzhou Ruixin Biotechnology Co., Ltd | RX103964H | Plasma | ng/mL | 0.125ng/mL-4ng/mL | <0.1 ng/mL | 1:5 | <10% | <15% |
| CTRP2 | Quanzhou Ruixin Biotechnology Co., Ltd | RX2D1015226 | Plasma | ng/mL | 0.625ng/mL-20ng/mL | <0.1 ng/mL | 1:5 | <10% | <15% |
| Visfatin | Quanzhou Ruixin Biotechnology Co., Ltd | RX105434H | Plasma | ng/mL | 3.75ng/mL-120ng/mL | <0.1 ng/mL | 1:5 | <10% | <15% |
| Vaspin | Quanzhou Ruixin Biotechnology Co., Ltd | RX104352H | Plasma | ng/mL | 0.375ng/mL-12ng/mL | <0.1 ng/mL | 1:5 | <10% | <15% |
| CTRP9 | ELK Biotechnology Co., Ltd | ELK10765 | Plasma | ng/mL | 3.13-200 ng/mL | 1.13 ng/mL | 1:10 | <8% | <10% |

Abbreviations: CV, coefficient of variation; CTRP, C1q/tumor necrosis factor-related protein; ELISA, enzyme-linked immunosorbent assay; LLOQ, lower limit of quantification; ROSC, return of spontaneous circulation.

**Table S3 - Patient baseline characteristics of the CT Imaging Cohort**

| **Variables** | **All patients (n=70)** |  |  | **Outcome** | |  |  |
| --- | --- | --- | --- | --- | --- | --- | --- |
|  |  | **Survivors (n=56)** | **Nonsurvivors (n=14)** | ***p***  **Value** | **Favourable**  **neurological**  **outcome (n=32)** | **Unfavourable**  **neurological**  **outcome (n=38)** | ***p***  **Value** |
| Age(y) (mean±SD†) | 56.19±17.47 | 54.31±16.6 | 63.07±19.43 | 0.085 | 51.81±18.14 | 59.84±16.21 | 0.054 |
| Male (%) | 49 (70.0%) | 39(70.9%) | 10(66.7%) | 0.490 | 23 (71.9%) | 26 (68.4%) | 0.753 |
| BMI | 23.09±4.43 | 23.06±4.52 | 23.19±4.25 | 0.919 | 23.51±4.58 | 22.73±4.34 | 0.472 |
| Initial rhythm-  Shockable | 15(21.4%) | 10(18.2%) | 5(33.3%) | 0.179 | 10 (31.3%) | 5 (13.2%) | 0.066 |
| **Comorbidities** |  |  |  |  |  |  |  |
| Heart disease (n, %) | 31 (44.2%) | 25 (44.6%) | 6 (42.9%) | 0.904 | 14 (43.8%) | 17(44.7%) | 0.934 |
| Respiratory  insufficiency (n, %) | 15 (21.4%) | 12 (21.8%) | 3 (20.0%) | 0.879 | 8 (25.0%) | 7 (18.4%) | 0.504 |
| Neurological  insufficiency (n, %) | 12 (17.1%) | 7 (12.7%) | 5 (33.3%) | 0.114 | 2 (6.3%) | 10 (26.3%) | 0.026* |
| Gastrointestinal  disease (n, %) | 7 (10.0%) | 6 (10.9%) | 1 (6.7%) | 0.627 | 2 (6.3%) | 5 (13.2%) | 0.442 |
| Renal insufficiency (n, %) | 6 (8.6%) | 5 (8.9%) | 1(6.7%) | 0.749 | 3 (9.4%) | 3(7.9%) | 0.462 |
| Sepsis‡ (n, %) | 11 (15.7%) | 10 (18.2%) | 1 (6.7%) | 0.435 | 3 (9.4%) | 8 (21.1%) | 0.181 |
| Metastatic  malignancy (n, %) | 5 (7.14%) | 3 (5.5%) | 2 (13.3%) | 0.290 | 0 | 5 (13.2%) | 0.058 |

**Note:** Categorical variables were compared using two-sided Fisher's exact tests. *p<0.05.

Abbreviations: †standard deviation;

‡Includes patients diagnosed with septic shock.

| **Variables** | **All patients (n=66)** |  |  | **Outcome** | |  |  |
| --- | --- | --- | --- | --- | --- | --- | --- |
|  |  | **Survivors (n=41)** | **Nonsurvivors (n=25)** | ***p***  **Value** | **Favourable**  **neurological**  **outcome (n=24)** | **Unfavourable**  **neurological**  **outcome (n=42)** | ***p***  **Value** |
| Age(y) (mean±SD)† | 60.71±14.26 | 60.37±16.21 | 61.28±10.61 | 0.783 | 59.08 ± 17.25 | 61.64 ± 12.37 | 0.487 |
| Male (%) | 48 (72.7%) | 31(75.6%) | 17(68.0%) | 0.574 | 19 (79.2%) | 29 (69.0%) | 0.566 |
| BMI | 22.78±5.02 | 22.93±4.93 | 22.53±5.26 | 0.758 | 23.06±5.04 | 22.62±5.06 | 0.735 |
| Initial rhythm-Shockable (n, %) | 17(25.8%) | 11(26.8%) | 6(24.0%) | 0.999 | 5 (20.8%) | 12 (28.6%) | 0.569 |
| **Comorbidities** |  |  |  |  |  |  |  |
| Heart disease (n, %) | 28 (42.4%) | 17 (41.5%) | 11(44.0%) | 0.998 | 11(45.8%) | 17(40.5%) | 0.797 |
| Respiratory  insufficiency (n, %) | 12 (18.2%) | 10 (24.4%) | 2 (8.0%) | 0.113 | 6 (25.0%) | 6 (14.3%) | 0.329 |
| Neurological  insufficiency (n, %) | 11 (16.7%) | 7 (17.1%) | 4 (16.0%) | 0.999 | 1 (4.2%) | 10 (23.8%) | 0.046* |
| Gastrointestinal  disease (n, %) | 4(6.1%) | 4(9.8%) | 0 | 0.289 | 2 (8.3%) | 2 (4.8%) | 0.618 |
| Renal insufficiency (n, %) | 10 (15.2%) | 7 (17.1%) | 3 (12.0%) | 0.730 | 4 (16.7%) | 6(14.3%) | 0.999 |
| Sepsis‡ (n, %) | 11 (16.7%) | 8 (19.5%) | 3 (12.0%) | 0.513 | 4(16.7%) | 7 (16.7%) | 0.997 |
| Malignancy (n, %) | 10(15.2%) | 4 (9.8%) | 6 (24.0%) | 0.161 | 1(4.16%) | 9 (21.4%) | 0.080 |

**Table S4 Patient baseline characteristics of the Biochemical Cohort.**

**Note:** Categorical variables were compared using two-sided Fisher's exact tests. *p<0.05.

| **Variables** | **Analyzed patients (n = 66)** | **Excluded because of severe hemolysis (n = 20)** | **p value** |
| --- | --- | --- | --- |
| Age, years | 60.71 ± 14.26 | 56.70 ± 16.73 | 0.340 |
| Male sex, n (%) | 48 (72.7%) | 16 (80.0%) | 0.770 |
| BMI, kg/m² | 22.78 ± 5.02 | 22.60 ± 3.09 | 0.846 |
| **Comorbidities** |  |  |  |
| Heart disease, n (%) | 28 (42.4%) | 8 (40.0%) | 0.999 |
| Respiratory insufficiency, n (%) | 12 (18.2%) | 3 (15.0%) | 0.999 |
| Neurological insufficiency, n (%) | 11 (16.7%) | 2 (10.0%) | 0.724 |
| Gastrointestinal disease, n (%) | 4 (6.1%) | 2 (10.0%) | 0.620 |
| Renal insufficiency, n (%) | 10 (15.2%) | 5 (25.0%) | 0.325 |
| Sepsis, n (%) | 11 (16.7%) | 2 (10.0%) | 0.724 |
| Malignancy, n (%) | 10 (15.2%) | 2 (10.0%) | 0.724 |
| **Cardiac arrest characteristics** |  |  |  |
| Initial shockable rhythm, n (%) | 17 (25.8%) | 4 (20.0%) | 0.769 |
| CPR duration, min | 9 (4–13) | 6.0 (3.5–12.0) | 0.513 |
| **Outcomes** |  |  |  |
| Survival to hospital discharge, n (%) | 41 (62.1%) | 16 (80.0%) | 0.181 |
| Favorable neurological outcome, n (%) | 24 (36.4%) | 5 (25.0%) | 0.425 |

**Table S5 Baseline characteristics of patients excluded because of severe hemolysis and those included in the Biochemical Cohort**

Note: Data are presented as mean ± SD, median (IQR), or n (%), as appropriate.

Continuous variables were compared using Welch’s t test (age and BMI) or the Mann–Whitney U test (CPR duration). Categorical variables were compared using Fisher’s exact test.

**Table S6 CPR data** **during inpatient grouped by survival at discharge**

| Variables | All patients (n=70) | Survivors (n=56) | Non-survivors (n=14) | p Value |
| --- | --- | --- | --- | --- |
| Total number of arrest (median, IQR) | 1(1) | 1(0) | 1(1) | 0.043* |
| Total time of CPR (median, IQR) | 6(11) | 6(11) | 5(17) | 0.926 |
| Abbreviations: IQR, interquartile range; CPR, cardiopulmonary resuscitation | | | | |

**Table S7 Time of tracheal intubation grouped by survival at discharge**

| Variables | All patients (n=70) | Survivors (n=56) | Non-survivors (n=14) | *p* Value |
| --- | --- | --- | --- | --- |
| Intubation, n (%) | 65(92.9%) | 51(92.7%) | 14(93.3%) | 0.936 |
| Intubate before CA, n (%) | 41(58.6%) | 31(56.4%) | 10(66.7%) | 0.473 |
| Intubate during CPR, n (%) | 35(50.0%) | 28(50.9%) | 7(46.7%) | 0.771 |
| Abbreviations: IQR, interquartile range; CA, cardiac arrest; CPR, cardiopulmonary resuscitation | | | | |

**Table S8 CPR data during inpatient grouped by** **neurological outcome at discharge**

| Variables | Studied population(n=58) | Favorable neurological outcome (n=30) | Unfavorable neurological outcome (n=28) | *p* Value |
| --- | --- | --- | --- | --- |
| Total number of arrest (median, IQR) | 1(1,2) | 1(1,2) | 1(1,1) | 0.275 |
| Total time of CPR (median, IQR) | 6.5(3,13) | 7(2,13) | 6.5(3.25,16.25) | 0.512 |
| Abbreviations: IQR, interquartile range; CPR, cardiopulmonary resuscitation | | | | |

**Table S9 Time of tracheal intubation grouped by neurological outcome at discharge**

| Variables | Studied population(n=58) | Favorable neurological outcome (n=30) | Unfavorable neurological outcome (n=28) | *p* Value |
| --- | --- | --- | --- | --- |
| Intubation, n (%) | 53(91.4%) | 25(83.3%) | 28(100.0%) | 0.053 |
| Intubate before CA, n (%) | 32(55.2%) | 11(36.7%) | 21(75.0%) | 0.003* |
| Intubate during CPR, n (%) | 30(51.7%) | 14(46.7%) | 16(57.1%) | 0.425 |
| Abbreviations: IQR, interquartile range; CA, cardiac arrest; CPR, cardiopulmonary resuscitation | | | | |

**Table S10 Time interval of recorded CT scan to CA and of ROSC to the rechecked CT scan stratified by survival at discharge**

| Variables | All patients (n=70) | Survivors (n=56) | Non-survivors (n=14) | *p* Value |
| --- | --- | --- | --- | --- |
| Time interval between the recorded CT and the first CA (d), (median, IQR) | 2.84(8.30) | 2.73(8.58) | 3.23(5.22) | 0.647 |
|  |  |  |  |  |
| Time interval between ROSC and the first rechecked CT(d), (median, IQR) | 6.35(9.23) | 6.28 (10.10) | 8.56(9.02) | 0.813 |
|  |  |  |  |  |
| Abbreviations: IQR, interquartile range; CT, computed tomography; CA, cardiac arrest; ROSC, return of spontaneous circulation | | | | |

**Table S11 Time interval of recorded CT scan to CA and of ROSC to the rechecked CT scan stratified by neurological outcome at discharge**

| Variables | Studied population(n=58) | Favorable neurological outcome (n=30) | Unfavorable neurological outcome (n=28) | *p* Value |
| --- | --- | --- | --- | --- |
| Time interval between the recorded CT and the first CA (d), (median, IQR) | 3.03(8.28) | 1.49(8.09) | 4.85(7.88) | 0.111 |
|  |  |  |  |  |
| Time interval between ROSC and the first rechecked CT(d), (median, IQR) | 7.59(13.30) | 9.69(15.03) | 5.61(7.71) | 0.056 |
|  |  |  |  |  |
| Abbreviations: IQR, interquartile range; CT, computed tomography; CA, cardiac arrest; ROSC, return of spontaneous circulation | | | | |

**Table S12 CPR data during the time interval of two CT scans stratified by survival at discharge**

| Variables | All patients (n=70) | Survivors (n=56) | Non-survivors (n=14) | *p* Value |
| --- | --- | --- | --- | --- |
| Total number of arrest (median, IQR) | 1(0.25) | 1(0) | 1(1) | 0.297 |
| Total time of CPR (median, IQR) | 6(9.5) | 6(11) | 6(11) | 0.994 |

**Table S13 CPR data during the time interval of two CT scans stratified by neurological outcome at discharge**

| Variables | Studied population(n=58) | Favorable neurological outcome (n=30) | Unfavorable neurological outcome (n=28) | *p* Value |
| --- | --- | --- | --- | --- |
| Total number of arrest (median, IQR) | 1(0.25) | 1(1) | 1(0) | 0.079 |
| Total time of CPR (median, IQR) | 6(10.25) | 6(11) | 6.5(10.25) | 0.408 |

Multiplicity correction for Tables S14-S37: Benjamini-Hochberg adjustment was applied separately within each combination of analysis population, CT timing, anatomical measure, and outcome. Each family comprised the 10 vertebral-level comparisons from T3 to T12.

**Table S14 Anterior SAT depth in each spinous process level measured among patients with different outcome at discharge in pre-arrest CT images**

| **aSAT (mm) (mean±SD)** | **Survivors (n=56)** | **Non-survivors (n=14)** | ***p* Value** | **BH q value** | **Favorable outcome (n=30)** | **Unfavorable outcome (n=28)** | ***p* Value** | **BH q value** |
| --- | --- | --- | --- | --- | --- | --- | --- | --- |
| T3 | 13.68±4.97 | 13.59±3.22 | 0.953 | 0.998 | 14.19±5.80 | 13.15±3.33 | 0.448 | 0.560 |
| T4 | 12.02±4.90 | 11.68±4.36 | 0.827 | 0.998 | 12.79±5.67 | 11.56±3.72 | 0.348 | 0.497 |
| T5 | 11.72±5.61 | 10.29±4.42 | 0.380 | 0.934 | 12.86±6.39 | 10.46±4.15 | 0.097 | 0.272 |
| T6 | 12.08±6.09 | 10.45±5.49 | 0.365 | 0.934 | 13.10±7.06 | 10.79±4.80 | 0.153 | 0.272 |
| T7 | 13.61±7.04 | 11.56±5.78 | 0.316 | 0.934 | 15.19±7.95 | 11.87±5.21 | 0.067 | 0.272 |
| T8 | 14.36±6.97 | 14.37±6.93 | 0.998 | 0.998 | 14.51±6.07 | 14.89±7.63 | 0.833 | 0.833 |
| T9 | 13.91±6.28 | 16.98±6.90 | 0.115 | 0.934 | 14.25±6.44 | 13.83±5.60 | 0.792 | 0.833 |
| T10 | 13.51±5.86 | 14.88±7.54 | 0.467 | 0.934 | 14.38±6.44 | 12.26±4.62 | 0.163 | 0.272 |
| T11 | 13.36±6.53 | 13.09±6.62 | 0.891 | 0.998 | 14.41±7.16 | 11.51±5.28 | 0.094 | 0.272 |
| T12 | 14.88±8.25 | 14.76±5.87 | 0.963 | 0.998 | 16.55±8.77 | 13.22±6.33 | 0.147 | 0.272 |
| Abbreviations: aSAT, anterior Subcutaneous Adipose Tissue; SD, standard deviation; BH q values were calculated separately across T3-T12 within each outcome using the Benjamini-Hochberg procedure; *q<0.05. | | | | | | | | |

**Table S15 Posterior SAT depth in each spinous process level measured among patients with different outcome at discharge in pre-arrest CT images**

| **pSAT (mm)**  **(mean±SD)** | **Survivors (n=56)** | **Non-survivors (n=14)** | ***p* Value** | **BH q value** | **Favorable outcome (n=30)** | **Unfavorable outcome (n=28)** | ***p* Value** | **BH q value** |
| --- | --- | --- | --- | --- | --- | --- | --- | --- |
| T3 | 22.72±10.20 | 21.58±7.77 | 0.716 | 0.975 | 23.15±9.21 | 22.11±10.38 | 0.697 | 0.697 |
| T4 | 21.31±8.51 | 19.20±5.87 | 0.385 | 0.975 | 22.05±8.33 | 19.66±7.40 | 0.257 | 0.286 |
| T5 | 19.54±7.25 | 18.37±4.74 | 0.571 | 0.975 | 20.15±7.79 | 17.93±5.66 | 0.222 | 0.278 |
| T6 | 19.33±7.32 | 17.38±5.26 | 0.353 | 0.975 | 20.25±7.98 | 16.92±5.91 | 0.075 | 0.179 |
| T7 | 18.55±7.91 | 18.01±5.87 | 0.812 | 0.975 | 19.72±8.74 | 16.39±6.48 | 0.103 | 0.179 |
| T8 | 18.50±8.42 | 17.48±5.99 | 0.671 | 0.975 | 19.89±9.23 | 16.12±7.02 | 0.085 | 0.179 |
| T9 | 18.58±9.24 | 18.48±5.53 | 0.959 | 0.975 | 20.38±10.00 | 15.78±7.10 | 0.053 | 0.179 |
| T10 | 18.49±9.78 | 18.75±6.52 | 0.907 | 0.975 | 20.23±10.23 | 16.23±8.46 | 0.115 | 0.179 |
| T11 | 18.63±10.53 | 18.56±6.41 | 0.975 | 0.975 | 20.49±11.02 | 16.18±9.01 | 0.118 | 0.179 |
| T12 | 17.24±10.97 | 18.84±6.19 | 0.644 | 0.975 | 19.54±11.83 | 14.97±8.04 | 0.125 | 0.179 |
| Abbreviations: pSAT, posterior subcutaneous adipose tissue depth; SD, standard deviation; BH q values were calculated separately across T3-T12 within each outcome using the Benjamini-Hochberg procedure; *q<0.05. | | | | | | | | |

**Table S16** **Cross-sectional in each vertebral level measured among patients with different outcome at discharge in pre-arrest CT images**

| **CsA (mm^2^)**  **(mean±SD)** | **Survivors (n=56)** | **Non-survivors (n=14)** | ***p* Value** | **BH q value** | **Favorable outcome (n=30)** | **Unfavorable outcome (n=28)** | ***p* Value** | **BH q value** |
| --- | --- | --- | --- | --- | --- | --- | --- | --- |
| T3 | 13648.89±4720.65 | 12780.20±3037.69 | 0.431 | 0.479 | 14270.16±5193.03 | 12914.23±4145.89 | 0.293 | 0.992 |
| T4 | 20321.50±5423.01 | 18792.42±4595.80 | 0.336 | 0.424 | 20868.96±5326.97 | 19442.31±5558.07 | 0.327 | 0.992 |
| T5 | 25726.34±5697.06 | 23559.65±5201.81 | 0.200 | 0.400 | 25548.25±5405.98 | 25265.80±6230.68 | 0.854 | 0.992 |
| T6 | 29839.57±6066.48 | 27283.19±5642.52 | 0.158 | 0.395 | 29385.37±5458.82 | 29487.04±6747.61 | 0.950 | 0.992 |
| T7 | 33062.55±6605.48 | 30121.11±6350.36 | 0.138 | 0.395 | 32537.10±5940.53 | 32672.18±7354.74 | 0.939 | 0.992 |
| T8 | 35156.98±7199.23 | 32033.13±6982.09 | 0.149 | 0.395 | 34646.37±6464.41 | 34665.13±8045.94 | 0.992 | 0.992 |
| T9 | 37008.91±7870.15 | 33626.15±7580.62 | 0.152 | 0.395 | 36565.86±7140.30 | 36525.78±8864.15 | 0.985 | 0.992 |
| T10 | 38261.91±8743.98 | 35249.76±8216.44 | 0.249 | 0.415 | 37864.43±7803.90 | 37958.25±9832.69 | 0.968 | 0.992 |
| T11 | 38945.32±9229.46 | 36318.12±8516.46 | 0.339 | 0.424 | 37901.23±7834.33 | 39454.67±10745.54 | 0.535 | 0.992 |
| T12 | 36872.96±8537.85 | 35525.22±7125.49 | 0.631 | 0.631 | 35926.88±7015.81 | 37615.15±9478.23 | 0.488 | 0.992 |
| Abbreviations: CsA, cross-sectional area; SD, standard deviation; BH q values were calculated separately across T3-T12 within each outcome using the Benjamini-Hochberg procedure; *q<0.05. | | | | | | | | |

**Table S17** **Transverse diameter in each vertebral level measured among patients with different outcome at discharge in pre-arrest CT images**

| **TD (mm)**  **(mean±SD)** | **Survivors (n=56)** | **Non-survivors (n=14)** | ***p* Value** | **BH q value** | **Favorable outcome (n=30)** | **Unfavorable outcome (n=28)** | ***p* Value** | **BH q value** |
| --- | --- | --- | --- | --- | --- | --- | --- | --- |
| T3 | 173.73±28.30 | 173.68±21.53 | 0.996 | 0.996 | 174.74±26.18 | 169.57±30.16 | 0.499 | 0.854 |
| T4 | 204.50±23.76 | 202.16±19.72 | 0.736 | 0.842 | 204.05±21.31 | 200.71±25.17 | 0.591 | 0.854 |
| T5 | 219.37±21.45 | 217.25±17.25 | 0.733 | 0.842 | 217.69±18.59 | 216.68±22.85 | 0.854 | 0.854 |
| T6 | 229.62±20.98 | 226.53±17.12 | 0.613 | 0.842 | 228.04±18.59 | 226.73±22.42 | 0.810 | 0.854 |
| T7 | 237.93±21.73 | 232.37±18.58 | 0.382 | 0.842 | 237.39±19.23 | 233.00±23.34 | 0.436 | 0.854 |
| T8 | 244.30±23.37 | 237.53±20.01 | 0.323 | 0.842 | 243.70±20.76 | 238.94±25.40 | 0.436 | 0.854 |
| T9 | 250.91±25.28 | 243.56±23.02 | 0.326 | 0.842 | 250.54±21.89 | 245.46±28.45 | 0.452 | 0.854 |
| T10 | 257.78±26.15 | 251.62±24.14 | 0.427 | 0.842 | 258.18±22.81 | 252.34±29.92 | 0.416 | 0.854 |
| T11 | 265.39±25.91 | 259.47±23.63 | 0.441 | 0.842 | 264.14±22.20 | 262.44±30.65 | 0.816 | 0.854 |
| T12 | 263.08±25.55 | 260.51±20.22 | 0.758 | 0.842 | 261.49±22.23 | 262.88±28.91 | 0.854 | 0.854 |
| Abbreviations: TD, transverse diameter; SD, standard deviation; BH q values were calculated separately across T3-T12 within each outcome using the Benjamini-Hochberg procedure; *q<0.05. | | | | | | | | |

**Table S18 Anteroposterior diameter in each vertebral level measured among patients with different outcome at discharge in pre-arrest CT images**

| **AD (mm)**  **(mean±SD)** | **Survivors (n=56)** | **Non-survivors (n=14)** | ***p* Value** | **BH q value** | **Favorable outcome (n=30)** | **Unfavorable outcome (n=28)** | ***p* Value** | **BH q value** |
| --- | --- | --- | --- | --- | --- | --- | --- | --- |
| T3 | 58.02±17.82 | 53.87±14.02 | 0.452 | 0.502 | 60.83±18.19 | 56.23±17.48 | 0.345 | 0.996 |
| T4 | 77.09±17.16 | 69.87±19.88 | 0.178 | 0.261 | 79.51±17.61 | 74.21±18.71 | 0.278 | 0.996 |
| T5 | 93.75±18.31 | 86.22±20.56 | 0.183 | 0.261 | 94.37±18.07 | 92.44±20.79 | 0.707 | 0.996 |
| T6 | 106.83±18.90 | 97.44±21.42 | 0.110 | 0.261 | 106.35±17.14 | 106.38±22.72 | 0.996 | 0.996 |
| T7 | 115.33±19.92 | 105.21±22.37 | 0.102 | 0.261 | 113.75±16.62 | 116.31±24.68 | 0.643 | 0.996 |
| T8 | 119.67±24.05 | 108.56±22.64 | 0.123 | 0.261 | 119.07±24.04 | 119.44±25.44 | 0.955 | 0.996 |
| T9 | 121.08±23.50 | 109.55±23.34 | 0.105 | 0.261 | 120.84±21.79 | 120.89±26.26 | 0.994 | 0.996 |
| T10 | 123.09±26.44 | 111.61±26.14 | 0.151 | 0.261 | 121.91±23.07 | 124.83±29.82 | 0.679 | 0.996 |
| T11 | 125.07±29.80 | 116.67±26.18 | 0.340 | 0.425 | 121.80±23.69 | 128.66±35.31 | 0.391 | 0.996 |
| T12 | 117.80±26.23 | 114.38±19.44 | 0.688 | 0.688 | 116.29±20.12 | 119.58±28.33 | 0.646 | 0.996 |
| Abbreviations: AD, anteroposterior diameter; SD, standard deviation; BH q values were calculated separately across T3-T12 within each outcome using the Benjamini-Hochberg procedure; *q<0.05. | | | | | | | | |

**Table S19** **Haller index in each vertebral level measured among patients with different outcome at discharge in pre-arrest CT images**

| **HI (mean±SD)** | **Survivors (n=56)** | **Non-survivors (n=14)** | ***p* Value** | **BH q value** | **Favorable outcome (n=30)** | **Unfavorable outcome (n=28)** | ***p* Value** | **BH q value** |
| --- | --- | --- | --- | --- | --- | --- | --- | --- |
| T3 | 3.16±0.72 | 3.40±0.80 | 0.316 | 0.395 | 2.98±0.62 | 3.23±0.81 | 0.213 | 0.903 |
| T4 | 2.74±0.56 | 3.12±0.95 | 0.172 | 0.394 | 2.63±0.53 | 2.88±0.8 | 0.194 | 0.903 |
| T5 | 2.42±0.47 | 2.64±0.58 | 0.135 | 0.394 | 2.37±0.44 | 2.47±0.58 | 0.476 | 0.903 |
| T6 | 2.21±0.39 | 2.42±0.48 | 0.102 | 0.394 | 2.19±0.33 | 2.24±0.50 | 0.647 | 0.903 |
| T7 | 2.12±0.37 | 2.29±0.42 | 0.158 | 0.394 | 2.13±0.30 | 2.11±0.46 | 0.83 | 0.903 |
| T8 | 2.12±0.40 | 2.26±0.42 | 0.246 | 0.394 | 2.12±0.36 | 2.11±0.47 | 0.895 | 0.903 |
| T9 | 2.15±0.39 | 2.30±0.44 | 0.221 | 0.394 | 2.14±0.34 | 2.15±0.47 | 0.903 | 0.903 |
| T10 | 2.19±0.46 | 2.34±0.46 | 0.276 | 0.394 | 2.19±0.37 | 2.17±0.56 | 0.875 | 0.903 |
| T11 | 2.23±0.49 | 2.31±0.44 | 0.618 | 0.687 | 2.25±0.40 | 2.21±0.60 | 0.795 | 0.903 |
| T12 | 2.33±0.49 | 2.32±0.28 | 0.932 | 0.932 | 2.31±0.34 | 2.35±0.60 | 0.768 | 0.903 |
| Abbreviations: HI, Haller index; SD, standard deviation; BH q values were calculated separately across T3-T12 within each outcome using the Benjamini-Hochberg procedure; *q<0.05. | | | | | | | | |

**Table S20 Anterior SAT depth in each spinous process level measured among patients with different outcome at discharge in post-arrest CT images**

| **aSAT (mm)**  **(mean±SD)** | **Survivors (n=56)** | **Non-survivors (n=14)** | ***p* Value** | **BH q value** | **Favorable outcome (n=30)** | **Unfavorable outcome (n=28)** | ***p* Value** | **BH q value** |
| --- | --- | --- | --- | --- | --- | --- | --- | --- |
| T3 | 12.69±5.97 | 13.69±2.91 | 0.595 | 0.996 | 13.54±6.50 | 12.36±5.08 | 0.498 | 0.659 |
| T4 | 11.09±4.92 | 11.08±4.28 | 0.996 | 0.996 | 12.16±5.60 | 10.40±4.29 | 0.209 | 0.659 |
| T5 | 10.70±4.81 | 11.13±5.44 | 0.769 | 0.996 | 11.32±5.57 | 10.46±4.61 | 0.530 | 0.659 |
| T6 | 11.36±5.69 | 11.23±6.13 | 0.940 | 0.996 | 12.15±6.53 | 10.79±5.24 | 0.386 | 0.659 |
| T7 | 13.01±6.47 | 12.32±6.86 | 0.725 | 0.996 | 14.03±7.17 | 12.35±5.90 | 0.335 | 0.659 |
| T8 | 13.68±6.85 | 14.86±7.89 | 0.579 | 0.996 | 14.34±7.13 | 13.69±6.23 | 0.712 | 0.712 |
| T9 | 13.68±6.81 | 17.24±6.43 | 0.082 | 0.820 | 14.53±7.53 | 13.58±5.84 | 0.593 | 0.659 |
| T10 | 12.72±6.06 | 14.45±7.38 | 0.366 | 0.996 | 13.65±7.26 | 11.45±4.74 | 0.175 | 0.659 |
| T11 | 12.37±6.14 | 13.89±8.87 | 0.475 | 0.996 | 13.43±7.25 | 11.52±4.85 | 0.245 | 0.659 |
| T12 | 13.67±7.42 | 14.02±6.76 | 0.886 | 0.996 | 14.49±7.84 | 12.95±6.17 | 0.471 | 0.659 |
| Abbreviations: aSAT, anterior subcutaneous adipose tissue depth; SD, standard deviation; BH q values were calculated separately across T3-T12 within each outcome using the Benjamini-Hochberg procedure; *q<0.05. | | | | | | | | |

**Table S21 Posterior SAT depth in each spinous process level measured among patients with different outcome at discharge in post-arrest CT images**

| **pSAT (mm)**  **(mean±SD)** | **Survivors (n=56)** | **Non-survivors (n=14)** | ***p* Value** | **BH q value** | **Favorable outcome (n=30)** | **Unfavorable outcome (n=28)** | ***p* Value** | **BH q value** |
| --- | --- | --- | --- | --- | --- | --- | --- | --- |
| T3 | 21.61±10.95 | 23.26±5.27 | 0.440 | 0.909 | 22.31±10.89 | 19.98±9.10 | 0.404 | 0.404 |
| T4 | 19.96±9.38 | 22.37±5.21 | 0.213 | 0.909 | 20.70±9.53 | 18.63±7.89 | 0.383 | 0.404 |
| T5 | 19.30±8.62 | 20.56±5.04 | 0.603 | 0.909 | 20.45±8.26 | 17.05±7.09 | 0.103 | 0.147 |
| T6 | 18.56±8.55 | 17.85±5.21 | 0.766 | 0.909 | 19.48±9.08 | 15.95±5.86 | 0.086 | 0.143 |
| T7 | 17.59±8.34 | 17.19±5.13 | 0.865 | 0.909 | 18.94±9.03 | 14.97±5.52 | 0.050 | 0.125 |
| T8 | 17.06±8.53 | 16.78±5.25 | 0.909 | 0.909 | 18.65±9.10 | 14.40±5.76 | 0.038 | 0.125 |
| T9 | 17.46±9.12 | 17.91±5.54 | 0.862 | 0.909 | 19.20±9.67 | 15.07±6.45 | 0.063 | 0.126 |
| T10 | 17.17±9.42 | 17.66±5.58 | 0.801 | 0.909 | 19.19±9.64 | 14.40±6.96 | 0.035 | 0.125 |
| T11 | 17.00±9.90 | 19.27±5.68 | 0.291 | 0.909 | 19.19±9.98 | 14.33±7.54 | 0.047 | 0.125 |
| T12 | 16.08±9.59 | 18.69±5.85 | 0.394 | 0.909 | 17.58±9.58 | 14.80±8.52 | 0.309 | 0.386 |
| Abbreviations: pSAT, posterior subcutaneous adipose tissue depth; SD, standard deviation; BH q values were calculated separately across T3-T12 within each outcome using the Benjamini-Hochberg procedure; *q<0.05. | | | | | | | | |

**Table S22 Cross-sectional in each vertebral level measured among patients with different outcome at discharge in post-arrest CT images**

| **CsA (mm^2^)**  **(mean±SD)** | **Survivors (n=56)** | **Non-survivors (n=14)** | ***p* Value** | **BH q value** | **Favorable outcome (n=30)** | **Unfavorable outcome (n=28)** | ***p* Value** | **BH q value** |
| --- | --- | --- | --- | --- | --- | --- | --- | --- |
| T3 | 13207.77±3892.11 | 13186.10±3296.06 | 0.985 | 0.985 | 13173.01±3869.11 | 13567.22±3804.79 | 0.711 | 0.866 |
| T4 | 19675.68±4830.51 | 19290.30±4626.31 | 0.790 | 0.894 | 19515.37±4529.96 | 19999.82±5166.23 | 0.713 | 0.866 |
| T5 | 25019.40±4950.63 | 24302.62±5332.47 | 0.635 | 0.894 | 24651.87±4312.14 | 25431.92±5828.09 | 0.565 | 0.866 |
| T6 | 29002.51±5316.49 | 28137.24±5681.91 | 0.593 | 0.894 | 28656.82±4561.06 | 29236.02±6124.25 | 0.686 | 0.866 |
| T7 | 32201.19±5815.43 | 31020.63±6025.62 | 0.502 | 0.894 | 31700.30±4787.41 | 32529.60±6812.95 | 0.597 | 0.866 |
| T8 | 34368.45±6320.39 | 32902.25±6282.68 | 0.440 | 0.894 | 33964.96±5366.26 | 34487.60±7130.03 | 0.755 | 0.866 |
| T9 | 36029.79±6910.27 | 34589.80±6567.03 | 0.484 | 0.894 | 35644.87±5937.87 | 36149.86±7633.60 | 0.779 | 0.866 |
| T10 | 37667.76±7812.42 | 35837.60±8117.23 | 0.439 | 0.894 | 37301.05±6960.53 | 37552.66±8825.35 | 0.904 | 0.904 |
| T11 | 38426.65±8193.04 | 36804.79±6689.36 | 0.524 | 0.894 | 37838.63±7317.89 | 38725.56±8693.89 | 0.680 | 0.866 |
| T12 | 36110.29±7708.81 | 36751.73±7531.31 | 0.805 | 0.894 | 36017.82±6754.21 | 37504.67±9174.67 | 0.531 | 0.866 |
| Abbreviations: CsA, cross-sectional area; SD, standard deviation; BH q values were calculated separately across T3-T12 within each outcome using the Benjamini-Hochberg procedure; *q<0.05. | | | | | | | | |

**Table S23 Transverse diameter in each vertebral level measured among patients with different outcome at discharge in post-arrest CT images**

| **TD (mm)**  **(mean±SD)** | **Survivors (n=56)** | **Non-survivors (n=14)** | ***p* Value** | **BH q value** | **Favorable outcome (n=30)** | **Unfavorable outcome (n=28)** | ***p* Value** | **BH q value** |
| --- | --- | --- | --- | --- | --- | --- | --- | --- |
| T3 | 173.73±28.30 | 173.68±21.53 | 0.996 | 0.996 | 13173.01±3869.11 | 13567.22±3804.79 | 0.711 | 0.866 |
| T4 | 204.50±23.76 | 202.16±19.72 | 0.736 | 0.842 | 19515.37±4529.96 | 19999.82±5166.23 | 0.713 | 0.866 |
| T5 | 219.37±21.45 | 217.25±17.25 | 0.733 | 0.842 | 24651.87±4312.14 | 25431.92±5828.09 | 0.565 | 0.866 |
| T6 | 229.62±20.98 | 226.53±17.12 | 0.613 | 0.842 | 28656.82±4561.06 | 29236.02±6124.25 | 0.686 | 0.866 |
| T7 | 237.93±21.73 | 232.37±18.58 | 0.382 | 0.842 | 31700.30±4787.41 | 32529.60±6812.95 | 0.597 | 0.866 |
| T8 | 244.30±23.37 | 237.53±20.01 | 0.323 | 0.842 | 33964.96±5366.26 | 34487.60±7130.03 | 0.755 | 0.866 |
| T9 | 250.91±25.28 | 243.56±23.02 | 0.326 | 0.842 | 35644.87±5937.87 | 36149.86±7633.60 | 0.779 | 0.866 |
| T10 | 257.78±26.15 | 251.62±24.14 | 0.427 | 0.842 | 37301.05±6960.53 | 37552.66±8825.35 | 0.904 | 0.904 |
| T11 | 265.39±25.91 | 259.47±23.63 | 0.441 | 0.842 | 37838.63±7317.89 | 38725.56±8693.89 | 0.680 | 0.866 |
| T12 | 263.08±25.55 | 260.51±20.22 | 0.758 | 0.842 | 36017.82±6754.21 | 37504.67±9174.67 | 0.531 | 0.866 |
| Abbreviations: TD, transverse diameter; SD, standard deviation; BH q values were calculated separately across T3-T12 within each outcome using the Benjamini-Hochberg procedure; *q<0.05. | | | | | | | | |

**Table S24** **Anteroposterior diameter in each vertebral level measured among patients with different outcome at discharge in post-arrest CT images**

| **AD (mm)**  **(mean±SD)** | **Survivors (n=56)** | **Non-survivors (n=14)** | ***p* Value** | **BH q value** | **Favorable outcome (n=30)** | **Unfavorable outcome (n=28)** | ***p* Value** | **BH q value** |
| --- | --- | --- | --- | --- | --- | --- | --- | --- |
| T3 | 55.45±14.72 | 63.07±30.51 | 0.195 | 0.687 | 56.82±13.28 | 56.02±15.46 | 0.842 | 0.905 |
| T4 | 73.95±17.55 | 69.59±17.72 | 0.412 | 0.687 | 74.59±16.50 | 75.56±18.35 | 0.841 | 0.905 |
| T5 | 91.06±17.43 | 86.23±19.60 | 0.372 | 0.687 | 91.67±15.20 | 92.26±20.23 | 0.905 | 0.905 |
| T6 | 102.15±18.19 | 99.12±18.89 | 0.581 | 0.766 | 101.29±15.61 | 105.24±20.38 | 0.425 | 0.905 |
| T7 | 109.94±18.26 | 107.96±18.76 | 0.718 | 0.798 | 108.00±14.88 | 113.76±21.51 | 0.255 | 0.905 |
| T8 | 115.60±21.85 | 109.86±17.18 | 0.365 | 0.687 | 114.03±21.42 | 117.19±22.28 | 0.596 | 0.905 |
| T9 | 115.91±20.78 | 110.56±17.73 | 0.380 | 0.687 | 114.76±18.70 | 116.75±22.32 | 0.724 | 0.905 |
| T10 | 119.62±24.38 | 113.67±21.10 | 0.406 | 0.687 | 118.97±23.38 | 120.30±24.32 | 0.838 | 0.905 |
| T11 | 119.38±24.07 | 115.59±20.03 | 0.613 | 0.766 | 117.27±22.14 | 120.53±24.75 | 0.618 | 0.905 |
| T12 | 113.54±21.26 | 115.24±23.24 | 0.816 | 0.816 | 114.27±17.28 | 116.45±27.17 | 0.754 | 0.905 |
| Abbreviations: AD, anteroposterior diameter; SD, standard deviation; BH q values were calculated separately across T3-T12 within each outcome using the Benjamini-Hochberg procedure; *q<0.05. | | | | | | | | |

**Table S25** **Haller index in each vertebral level measured among patients with different outcome at discharge in post-arrest CT images**

| **HI**  **(mean±SD)** | **Survivors (n=56)** | **Non-survivors (n=14)** | ***p* Value** | **BH q value** | **Favorable outcome (n=30)** | **Unfavorable outcome (n=28)** | ***p* Value** | **BH q value** |
| --- | --- | --- | --- | --- | --- | --- | --- | --- |
| T3 | 3.03±0.70 | 3.16±0.21 | 0.729 | 0.755 | 3.13±0.53 | 3.30±0.69 | 0.333 | 0.954 |
| T4 | 2.68±0.60 | 2.78±0.33 | 0.755 | 0.755 | 2.79±0.53 | 2.85±0.62 | 0.720 | 0.954 |
| T5 | 2.36±0.49 | 2.65±0.33 | 0.256 | 0.366 | 2.42±0.37 | 2.50±0.54 | 0.512 | 0.954 |
| T6 | 2.17±0.39 | 2.43±0.27 | 0.212 | 0.366 | 2.29±0.32 | 2.27±0.44 | 0.854 | 0.954 |
| T7 | 2.10±0.38 | 2.35±0.26 | 0.201 | 0.366 | 2.22±0.27 | 2.16±0.40 | 0.543 | 0.954 |
| T8 | 2.10±0.42 | 2.37±0.35 | 0.222 | 0.366 | 2.18±0.31 | 2.16±0.41 | 0.865 | 0.954 |
| T9 | 2.14±0.39 | 2.38±0.35 | 0.250 | 0.366 | 2.22±0.30 | 2.22±0.42 | 0.954 | 0.954 |
| T10 | 2.17±0.47 | 2.47±0.28 | 0.225 | 0.366 | 2.22±0.35 | 2.24±0.48 | 0.834 | 0.954 |
| T11 | 2.20±0.48 | 2.53±0.54 | 0.202 | 0.366 | 2.29±0.37 | 2.31±0.48 | 0.882 | 0.954 |
| T12 | 2.27±0.43 | 2.37±0.09 | 0.748 | 0.755 | 2.31±0.29 | 2.42±0.55 | 0.425 | 0.954 |
| Abbreviations: HI, Haller index; SD, standard deviation; BH q values were calculated separately across T3-T12 within each outcome using the Benjamini-Hochberg procedure; *q<0.05. | | | | | | | | |

**Table S26 Anterior SAT depth in each spinous process level in pre-arrest CT images measured among patients receiving same kind of ventilation support when performing two CT scans**

| **aSAT (mm) (mean±SD)** | **Survivors (n=41)** | **Non-survivors (n=4)** | ***p* Value** | **BH q value** | **Favorable outcome (n=27)** | **Unfavorable outcome (n=12)** | ***p* Value** | **BH q value** |
| --- | --- | --- | --- | --- | --- | --- | --- | --- |
| T3 | 14.23±5.21 | 14.44±3.95 | 0.937 | 0.937 | 14.51±5.94 | 14.08±2.67 | 0.776 | 0.779 |
| T4 | 12.46±5.15 | 11.78±5.55 | 0.802 | 0.891 | 12.95±5.84 | 12.43±3.44 | 0.779 | 0.779 |
| T5 | 12.33±5.84 | 9.89±3.91 | 0.421 | 0.774 | 13.44±6.45 | 10.76±3.57 | 0.184 | 0.391 |
| T6 | 12.56±6.45 | 10.4±4.75 | 0.518 | 0.774 | 13.66±7.18 | 10.91±4.31 | 0.227 | 0.391 |
| T7 | 14.66±7.34 | 10.96±4.84 | 0.331 | 0.774 | 15.90±7.99 | 13.12±5.01 | 0.274 | 0.391 |
| T8 | 15.27±6.62 | 13.1±7.21 | 0.538 | 0.774 | 14.81±5.76 | 17.44±7.78 | 0.246 | 0.391 |
| T9 | 14.61±6.64 | 14.98±0.73 | 0.741 | 0.891 | 14.56±6.55 | 13.75±4.48 | 0.710 | 0.779 |
| T10 | 14.25±6.26 | 13.06±1.47 | 0.348 | 0.774 | 14.82±6.57 | 12.06±3.88 | 0.203 | 0.391 |
| T11 | 13.89±6.91 | 10.02±3.94 | 0.146 | 0.774 | 14.50±7.48 | 11.01±4.14 | 0.174 | 0.391 |
| T12 | 15.87±8.49 | 12.11±3.63 | 0.542 | 0.774 | 17.23±9.05 | 12.38±3.98 | 0.157 | 0.391 |
| Abbreviations: aSAT, anterior Subcutaneous Adipose Tissue; SD, standard deviation; BH q values were calculated separately across T3-T12 within each outcome using the Benjamini-Hochberg procedure; *q<0.05. | | | | | | | | |

**Table S27 Posterior SAT depth in each spinous process level in pre-arrest CT images measured among patients receiving same kind of ventilation support when performing two CT scans**

| **pSAT (mm)**  **(mean±SD)** | **Survivors (n=41)** | **Non-survivors (n=4)** | ***p* Value** | **BH q value** | **Favorable outcome (n=27)** | **Unfavorable outcome (n=12)** | ***p* Value** | **BH q value** |
| --- | --- | --- | --- | --- | --- | --- | --- | --- |
| T3 | 22.92±9.47 | 27.57±6.88 | 0.346 | 0.902 | 23.86±9.15 | 22.21±8.92 | 0.602 | 0.602 |
| T4 | 22.02±8.35 | 25.15±5.72 | 0.469 | 0.902 | 22.83±8.10 | 20.92±6.65 | 0.478 | 0.531 |
| T5 | 20.33±7.41 | 21.12±3.35 | 0.837 | 0.902 | 21.23±7.47 | 18.26±5.15 | 0.220 | 0.275 |
| T6 | 20.28±7.44 | 18.54±2.84 | 0.646 | 0.902 | 21.22±7.82 | 17.19±4.03 | 0.041 | 0.068 |
| T7 | 19.69±7.63 | 19.05±2.03 | 0.868 | 0.902 | 20.92±8.32 | 16.64±3.41 | 0.029 | 0.058 |
| T8 | 19.58±8.10 | 18.65±3.84 | 0.822 | 0.902 | 21.05±8.91 | 16.21±3.32 | 0.018 | 0.045* |
| T9 | 19.68±9.24 | 19.10±2.49 | 0.766 | 0.902 | 21.44±9.87 | 15.48±4.15 | 0.013 | 0.045* |
| T10 | 19.62±9.42 | 19.03±3.68 | 0.902 | 0.902 | 21.37±10.05 | 15.65±3.59 | 0.014 | 0.045* |
| T11 | 19.78±10.17 | 18.99±2.81 | 0.719 | 0.902 | 21.72±10.82 | 15.43±3.58 | 0.012 | 0.045* |
| T12 | 19.08±11.04 | 20.57±3.58 | 0.677 | 0.902 | 20.81±11.91 | 14.99±4.94 | 0.069 | 0.099 |
| Abbreviations: pSAT, posterior subcutaneous adipose tissue depth; SD, standard deviation; BH q values were calculated separately across T3-T12 within each outcome using the Benjamini-Hochberg procedure; *q<0.05. | | | | | | | | |

**Table S28 Cross-sectional in each vertebral level in pre-arrest CT images measured among patients receiving same kind of ventilation support when performing two CT scans**

| **CsA (mm^2^)**  **(mean±SD)** | **Survivors (n=41)** | **Non-survivors (n=4)** | ***p* Value** | **BH q value** | **Favorable outcome (n=27)** | **Unfavorable outcome (n=12)** | ***p* Value** | **BH q value** |
| --- | --- | --- | --- | --- | --- | --- | --- | --- |
| T3 | 13944.76±4941.13 | 13738.40±2859.35 | 0.935 | 0.949 | 14416.29±5353.05 | 12785.00±4219.54 | 0.357 | 0.707 |
| T4 | 20568.97±5537.59 | 20388.10±3018.77 | 0.949 | 0.949 | 21075.12±5469.80 | 19035.47±5680.13 | 0.295 | 0.707 |
| T5 | 26115.25±5625.32 | 24257.22±4301.11 | 0.526 | 0.751 | 26096.57±5375.38 | 24947.56±6436.75 | 0.566 | 0.707 |
| T6 | 30089.33±5878.34 | 27568.42±4230.76 | 0.410 | 0.695 | 29895.15±5520.58 | 28704.33±6444.73 | 0.558 | 0.707 |
| T7 | 33235.07±6451.61 | 30341.64±4930.25 | 0.390 | 0.695 | 33055.41±6041.95 | 31650.27±7081.94 | 0.529 | 0.707 |
| T8 | 35411.94±7018.31 | 32183.29±5863.85 | 0.380 | 0.695 | 35289.53±6467.69 | 33570.47±7938.00 | 0.480 | 0.707 |
| T9 | 37222.92±7559.61 | 33694.63±6646.75 | 0.374 | 0.695 | 37248.10±7123.55 | 35282.97±8784.04 | 0.464 | 0.707 |
| T10 | 38375.95±8149.97 | 34893.13±7474.71 | 0.417 | 0.695 | 38643.52±7678.45 | 35811.47±9414.82 | 0.341 | 0.707 |
| T11 | 39093.55±8376.33 | 35370.74±8166.29 | 0.401 | 0.695 | 38798.63±7545.50 | 37474.44±10772.83 | 0.676 | 0.751 |
| T12 | 37430.02±8097.80 | 36268.58±1657.41 | 0.843 | 0.949 | 36986.10±6541.37 | 36467.21±10568.00 | 0.872 | 0.872 |
| Abbreviations: CsA, cross-sectional area; SD, standard deviation; BH q values were calculated separately across T3-T12 within each outcome using the Benjamini-Hochberg procedure; *q<0.05. | | | | | | | | |

**Table S29 Transverse diameter in each vertebral level in pre-arrest CT images measured among patients receiving same kind of ventilation support when performing two CT scans**

| **TD (mm)**  **(mean±SD)** | **Survivors (n=41)** | **Non-survivors (n=4)** | ***p* Value** | **BH q value** | **Favorable outcome (n=27)** | **Unfavorable outcome (n=12)** | ***p* Value** | **BH q value** |
| --- | --- | --- | --- | --- | --- | --- | --- | --- |
| T3 | 174.40±27.57 | 183.38±22.34 | 0.533 | 0.965 | 174.34±27.10 | 168.39±27.45 | 0.533 | 0.533 |
| T4 | 204.25±23.18 | 210.05±21.05 | 0.633 | 0.965 | 203.94±22.07 | 197.15±22.18 | 0.382 | 0.503 |
| T5 | 219.65±20.93 | 223.39±18.23 | 0.733 | 0.965 | 218.57±19.20 | 212.83±20.21 | 0.402 | 0.503 |
| T6 | 229.78±20.28 | 231.92±15.85 | 0.839 | 0.965 | 228.81±19.47 | 222.80±17.72 | 0.367 | 0.503 |
| T7 | 238.01±20.80 | 239.04±17.71 | 0.924 | 0.965 | 238.05±20.05 | 228.77±17.82 | 0.177 | 0.503 |
| T8 | 244.13±22.12 | 243.29±18.26 | 0.942 | 0.965 | 244.57±21.48 | 234.67±20.14 | 0.184 | 0.503 |
| T9 | 251.04±23.49 | 249.09±18.56 | 0.873 | 0.965 | 251.83±22.51 | 242.19±23.97 | 0.234 | 0.503 |
| T10 | 258.25±24.29 | 258.80±21.61 | 0.965 | 0.965 | 259.91±23.03 | 247.44±25.56 | 0.151 | 0.503 |
| T11 | 265.63±24.21 | 263.91±22.91 | 0.893 | 0.965 | 265.99±21.92 | 257.01±29.65 | 0.322 | 0.503 |
| T12 | 264.03±24.21 | 270.15±4.94 | 0.727 | 0.965 | 264.15±21.34 | 257.73±30.89 | 0.523 | 0.533 |
| Abbreviations: TD, transverse diameter; SD, standard deviation; BH q values were calculated separately across T3-T12 within each outcome using the Benjamini-Hochberg procedure; *q<0.05. | | | | | | | | |

**Table S30** **Anteroposterior diameter in each vertebral level in pre-arrest CT images measured among patients receiving same kind of ventilation support when performing two CT scans**

| **AD (mm)**  **(mean±SD)** | **Survivors (n=41)** | **Non-survivors (n=4)** | ***p* Value** | **BH q value** | **Favorable outcome (n=27)** | **Unfavorable outcome (n=12)** | ***p* Value** | **BH q value** |
| --- | --- | --- | --- | --- | --- | --- | --- | --- |
| T3 | 60.23±18.33 | 58.01±5.16 | 0.576 | 0.720 | 61.81±18.48 | 58.87±18.98 | 0.652 | 0.897 |
| T4 | 78.72±17.79 | 76.03±9.02 | 0.769 | 0.769 | 80.52±17.84 | 76.84±18.20 | 0.557 | 0.897 |
| T5 | 95.81±18.13 | 85.81±15.98 | 0.295 | 0.421 | 96.57±17.60 | 93.26±21.58 | 0.616 | 0.897 |
| T6 | 108.62±18.10 | 96.66±15.68 | 0.210 | 0.370 | 108.26±16.96 | 107.4±22.85 | 0.897 | 0.897 |
| T7 | 116.91±19.50 | 103.01±17.56 | 0.178 | 0.370 | 115.45±16.67 | 118.05±26.70 | 0.712 | 0.897 |
| T8 | 121.53±24.58 | 105.10±20.80 | 0.204 | 0.370 | 121.06±24.51 | 119.80±28.31 | 0.888 | 0.897 |
| T9 | 122.31±23.21 | 107.00±20.69 | 0.212 | 0.370 | 122.45±21.82 | 119.58±29.73 | 0.736 | 0.897 |
| T10 | 124.60±24.89 | 106.54±19.19 | 0.168 | 0.370 | 124.26±22.27 | 122.72±32.93 | 0.867 | 0.897 |
| T11 | 127.14±28.64 | 108.57±27.22 | 0.222 | 0.370 | 124.53±22.22 | 128.25±45.35 | 0.809 | 0.897 |
| T12 | 121.04±24.24 | 114.21±6.27 | 0.697 | 0.769 | 119.41±17.82 | 118.04±34.33 | 0.887 | 0.897 |
| Abbreviations: AD, anteroposterior diameter; SD, standard deviation; BH q values were calculated separately across T3-T12 within each outcome using the Benjamini-Hochberg procedure; *q<0.05. | | | | | | | | |

**Table S31 Haller index in each vertebral level** **in pre-arrest CT images measured among patients receiving same kind of ventilation support when performing two CT scans**

| **HI (mean±SD)** | **Survivors (n=41)** | **Non-survivors (n=4)** | ***p* Value** | **BH q value** | **Favorable outcome (n=27)** | **Unfavorable outcome (n=12)** | ***p* Value** | **BH q value** |
| --- | --- | --- | --- | --- | --- | --- | --- | --- |
| T3 | 3.03±0.70 | 3.16±0.21 | 0.729 | 0.755 | 2.91±0.59 | 3.03±0.70 | 0.621 | 0.891 |
| T4 | 2.68±0.60 | 2.78±0.33 | 0.755 | 0.755 | 2.59±0.53 | 2.68±0.67 | 0.691 | 0.891 |
| T5 | 2.36±0.49 | 2.65±0.33 | 0.256 | 0.366 | 2.31±0.43 | 2.40±0.58 | 0.634 | 0.891 |
| T6 | 2.17±0.39 | 2.43±0.27 | 0.212 | 0.366 | 2.15±0.33 | 2.18±0.51 | 0.837 | 0.930 |
| T7 | 2.10±0.38 | 2.35±0.26 | 0.201 | 0.366 | 2.10±0.31 | 2.05±0.50 | 0.713 | 0.891 |
| T8 | 2.10±0.42 | 2.37±0.35 | 0.222 | 0.366 | 2.10±0.37 | 2.10±0.55 | 0.996 | 0.996 |
| T9 | 2.14±0.39 | 2.38±0.35 | 0.250 | 0.366 | 2.12±0.34 | 2.19±0.54 | 0.669 | 0.891 |
| T10 | 2.17±0.47 | 2.47±0.28 | 0.225 | 0.366 | 2.15±0.36 | 2.22±0.70 | 0.712 | 0.891 |
| T11 | 2.20±0.48 | 2.53±0.54 | 0.202 | 0.366 | 2.20±0.37 | 2.28±0.83 | 0.691 | 0.891 |
| T12 | 2.27±0.43 | 2.37±0.09 | 0.748 | 0.755 | 2.25±0.27 | 2.40±0.75 | 0.443 | 0.891 |

Abbreviations: HI, Haller index; SD, standard deviation; BH q values were calculated separately across T3-T12 within each outcome using the Benjamini-Hochberg procedure; *q<0.05.

**Table S32 Anterior SAT depth in each spinous process level in post-arrest CT images measured among patients receiving same kind of ventilation support when performing two CT scans**

| **aSAT (mm)**  **(mean±SD)** | **Survivors (n=41)** | **Non-survivors (n=4)** | ***p* Value** | **BH q value** | **Favorable outcome (n=27)** | **Unfavorable outcome (n=12)** | ***p* Value** | **BH q value** |
| --- | --- | --- | --- | --- | --- | --- | --- | --- |
| T3 | 13.20±5.89 | 14.26±3.12 | 0.728 | 0.957 | 13.80±6.75 | 12.60±2.69 | 0.480 | 0.819 |
| T4 | 11.46±5.20 | 12.62±6.59 | 0.679 | 0.957 | 12.29±5.79 | 11.00±4.71 | 0.519 | 0.819 |
| T5 | 11.11±4.95 | 12.47±7.31 | 0.617 | 0.957 | 11.78±5.61 | 10.92±4.68 | 0.655 | 0.819 |
| T6 | 11.59±5.86 | 12.18±7.13 | 0.852 | 0.957 | 12.60±6.65 | 10.72±4.57 | 0.380 | 0.819 |
| T7 | 13.65±6.50 | 13.03±8.57 | 0.861 | 0.957 | 14.66±7.16 | 13.33±5.52 | 0.573 | 0.819 |
| T8 | 14.40±6.96 | 13.42±8.21 | 0.792 | 0.957 | 14.89±7.16 | 14.48±5.23 | 0.859 | 0.859 |
| T9 | 14.44±7.35 | 16.85±3.62 | 0.523 | 0.957 | 14.95±7.60 | 14.40±5.80 | 0.825 | 0.859 |
| T10 | 13.45±6.47 | 13.52±3.80 | 0.984 | 0.984 | 14.12±7.38 | 11.50±3.90 | 0.256 | 0.819 |
| T11 | 12.81±6.76 | 9.04±3.59 | 0.347 | 0.957 | 13.71±7.58 | 10.44±3.55 | 0.080 | 0.800 |
| T12 | 14.14±8.09 | 11.13±3.49 | 0.608 | 0.957 | 14.61±8.19 | 12.13±4.42 | 0.455 | 0.819 |
| Abbreviations: aSAT, anterior subcutaneous adipose tissue depth; SD, standard deviation; BH q values were calculated separately across T3-T12 within each outcome using the Benjamini-Hochberg procedure; *q<0.05. | | | | | | | | |

**Table S33 Posterior SAT depth in each spinous process level in post-arrest CT images measured among patients receiving same kind of ventilation support when performing two CT scans**

| **pSAT (mm)**  **(mean±SD)** | **Survivors (n=41)** | **Non-survivors (n=4)** | ***p* Value** | **BH q value** | **Favorable outcome (n=27)** | **Unfavorable outcome (n=12)** | ***p* Value** | **BH q value** |
| --- | --- | --- | --- | --- | --- | --- | --- | --- |
| T3 | 21.76±10.76 | 23.43±3.49 | 0.513 | 0.747 | 22.99±10.95 | 18.21±6.99 | 0.193 | 0.214 |
| T4 | 20.52±9.12 | 23.95±3.46 | 0.463 | 0.747 | 21.56±9.31 | 17.95±6.61 | 0.252 | 0.252 |
| T5 | 20.13±8.35 | 21.42±5.18 | 0.765 | 0.765 | 21.38±8.13 | 16.85±6.95 | 0.114 | 0.151 |
| T6 | 19.36±8.73 | 17.66±4.43 | 0.704 | 0.765 | 20.52±8.95 | 15.96±6.40 | 0.121 | 0.151 |
| T7 | 18.34±8.61 | 15.71±2.32 | 0.550 | 0.747 | 19.91±9.00 | 14.21±4.76 | 0.047 | 0.078 |
| T8 | 17.88±8.71 | 15.26±3.60 | 0.558 | 0.747 | 19.68±8.96 | 13.44±4.51 | 0.006 | 0.014* |
| T9 | 18.16±9.24 | 15.67±3.85 | 0.598 | 0.747 | 20.30±9.51 | 13.62±4.91 | 0.007 | 0.014* |
| T10 | 17.61±9.39 | 15.53±2.56 | 0.303 | 0.747 | 20.30±9.50 | 11.87±3.43 | <0.001 | <0.001* |
| T11 | 17.52±9.85 | 14.80±1.86 | 0.167 | 0.747 | 20.35±9.81 | 10.82±3.08 | <0.001 | <0.001* |
| T12 | 16.92±9.57 | 13.56±0.21 | 0.060 | 0.600 | 18.71±9.56 | 10.73±2.86 | 0.002 | 0.007* |
| Abbreviations: pSAT, posterior subcutaneous adipose tissue depth; SD, standard deviation; BH q values were calculated separately across T3-T12 within each outcome using the Benjamini-Hochberg procedure; *q<0.05. | | | | | | | | |

**Table S34 Cross-sectional in each vertebral level in post-arrest CT images measured among patients receiving same kind of ventilation support when performing two CT scans**

| **CsA (mm^2^)**  **(mean±SD)** | **Survivors (n=41)** | **Non-survivors (n=4)** | ***p* Value** | **BH q value** | **Favorable outcome (n=27)** | **Unfavorable outcome (n=12)** | ***p* Value** | **BH q value** |
| --- | --- | --- | --- | --- | --- | --- | --- | --- |
| T3 | 13360.63±4018.68 | 12365.76±1552.38 | 0.352 | 0.943 | 13295.59±3992.20 | 13340.88±3974.47 | 0.975 | 0.975 |
| T4 | 20161.59±4673.82 | 19463.40±1877.22 | 0.577 | 0.943 | 19914.13±4362.32 | 20533.33±4950.43 | 0.707 | 0.975 |
| T5 | 25475.05±4803.98 | 24948.18±1442.65 | 0.624 | 0.943 | 25131.00±4221.01 | 25829.42±5643.40 | 0.678 | 0.975 |
| T6 | 29338.12±5146.34 | 29129.35±1082.28 | 0.831 | 0.943 | 29163.93±4516.80 | 28869.24±5732.62 | 0.864 | 0.975 |
| T7 | 32395.15±5630.46 | 32191.05±1371.22 | 0.943 | 0.943 | 32168.23±4812.58 | 31991.33±6644.84 | 0.926 | 0.975 |
| T8 | 34586.67±6182.95 | 34204.30±2112.90 | 0.904 | 0.943 | 34457.60±5392.46 | 33848.55±7061.79 | 0.769 | 0.975 |
| T9 | 36244.53±6786.25 | 35939.78±2429.33 | 0.930 | 0.943 | 36173.73±5911.44 | 35382.82±7725.32 | 0.728 | 0.975 |
| T10 | 38025.22±7684.80 | 38195.69±2339.91 | 0.921 | 0.943 | 38032.97±6835.14 | 37132.58±8667.98 | 0.729 | 0.975 |
| T11 | 38784.70±7998.11 | 40290.58±1804.05 | 0.375 | 0.943 | 38701.78±7070.87 | 38355.74±9612.52 | 0.903 | 0.975 |
| T12 | 36336.52±7209.16 | 38944.15±57.08 | 0.618 | 0.943 | 36958.71±6454.68 | 34683.02±9313.63 | 0.472 | 0.975 |
| Abbreviations: CsA, cross-sectional area; SD, standard deviation; BH q values were calculated separately across T3-T12 within each outcome using the Benjamini-Hochberg procedure; *q<0.05. | | | | | | | | |

**Table S35 Transverse diameter in each vertebral level in post-arrest CT images measured among patients receiving same kind of ventilation support when performing two CT scans**

| **TD (mm)**  **(mean±SD)** | **Survivors (n=41)** | **Non-survivors (n=4)** | ***p* Value** | **BH q value** | **Favorable outcome (n=27)** | **Unfavorable outcome (n=12)** | ***p* Value** | **BH q value** |
| --- | --- | --- | --- | --- | --- | --- | --- | --- |
| T3 | 174.06±23.74 | 177.73±19.69 | 0.769 | 0.909 | 172.65±22.95 | 174.30±23.99 | 0.845 | 0.924 |
| T4 | 204.67±18.74 | 206.59±18.74 | 0.848 | 0.909 | 202.49±17.56 | 203.85±18.87 | 0.835 | 0.924 |
| T5 | 220.57±18.17 | 225.49±12.93 | 0.603 | 0.909 | 218.36±16.65 | 218.93±16.46 | 0.924 | 0.924 |
| T6 | 231.22±17.99 | 234.99±9.44 | 0.685 | 0.909 | 229.65±17.03 | 224.76±17.64 | 0.418 | 0.924 |
| T7 | 238.67±18.28 | 243.80±5.32 | 0.220 | 0.909 | 237.59±17.16 | 232.78±18.08 | 0.432 | 0.924 |
| T8 | 245.04±20.35 | 250.14±3.02 | 0.168 | 0.909 | 243.89±18.60 | 239.21±20.51 | 0.487 | 0.924 |
| T9 | 251.91±21.68 | 257.69±6.38 | 0.602 | 0.909 | 251.20±20.54 | 247.71±22.51 | 0.637 | 0.924 |
| T10 | 260.00±23.08 | 268.92±9.32 | 0.452 | 0.909 | 259.40±21.13 | 255.82±25.57 | 0.650 | 0.924 |
| T11 | 265.80±24.01 | 279.33±2.78 | 0.434 | 0.909 | 264.83±22.23 | 262.96±28.21 | 0.829 | 0.924 |
| T12 | 263.13±22.22 | 272.54±0.97 | 0.909 | 0.909 | 263.84±20.38 | 257.44±28.50 | 0.516 | 0.924 |
| Abbreviations: TD, transverse diameter; SD, standard deviation; BH q values were calculated separately across T3-T12 within each outcome using the Benjamini-Hochberg procedure; *q<0.05. | | | | | | | | |

**Table S36 Anteroposterior diameter in each vertebral level in post-arrest CT images measured among patients receiving same kind of ventilation support when performing two CT scans**

| **AD (mm)**  **(mean±SD)** | **Survivors (n=41)** | **Non-survivors (n=4)** | ***p* Value** | **BH q value** | **Favorable outcome (n=27)** | **Unfavorable outcome (n=12)** | ***p* Value** | **BH q value** |
| --- | --- | --- | --- | --- | --- | --- | --- | --- |
| T3 | 57.32±15.71 | 54.34±1.04 | 0.264 | 0.718 | 57.28±14.05 | 56.59±16.87 | 0.889 | 0.976 |
| T4 | 76.38±18.31 | 72.47±3.34 | 0.266 | 0.718 | 75.53±17.21 | 76.79±18.40 | 0.827 | 0.976 |
| T5 | 92.83±17.82 | 87.45±5.15 | 0.187 | 0.718 | 92.26±16.40 | 92.42±18.80 | 0.976 | 0.976 |
| T6 | 103.76±17.75 | 100.76±2.63 | 0.349 | 0.718 | 102.82±16.78 | 104.66±17.64 | 0.740 | 0.976 |
| T7 | 110.77±18.46 | 107.18±4.90 | 0.704 | 0.880 | 109.27±16.65 | 112.51±19.68 | 0.574 | 0.976 |
| T8 | 116.36±23.32 | 111.81±6.89 | 0.702 | 0.880 | 114.94±22.77 | 117.71±22.00 | 0.704 | 0.976 |
| T9 | 115.95±21.10 | 112.2±4.20 | 0.359 | 0.718 | 115.12±19.43 | 116.44±21.94 | 0.841 | 0.976 |
| T10 | 119.85±24.59 | 115.95±5.68 | 0.436 | 0.727 | 118.79±22.99 | 120.71±25.00 | 0.803 | 0.976 |
| T11 | 120.22±24.59 | 121.93±8.31 | 0.794 | 0.882 | 118.79±22.33 | 123.34±26.85 | 0.568 | 0.976 |
| T12 | 113.77±21.47 | 115.62±2.38 | 0.906 | 0.906 | 114.53±18.39 | 112.14±27.58 | 0.785 | 0.976 |

Abbreviations: AD, anteroposterior diameter; SD, standard deviation; BH q values were calculated separately across T3-T12 within each outcome using the Benjamini-Hochberg procedure; *q<0.05.

**Table S37 Haller index in each vertebral level in post-arrest CT images measured among patients receiving same kind of ventilation support when performing two CT scans**

| **HI (mean±SD)** | **Survivors (n=56)** | **Non-survivors (n=14)** | ***p* Value** | **BH q value** | **Favorable outcome (n=27)** | **Unfavorable outcome (n=12)** | ***p* Value** | **BH q value** |
| --- | --- | --- | --- | --- | --- | --- | --- | --- |
| T3 | 3.19±0.68 | 3.27±0.36 | 0.818 | 0.983 | 3.16±0.61 | 3.27±0.74 | 0.589 | 0.988 |
| T4 | 2.82±0.68 | 2.85±0.28 | 0.979 | 0.983 | 2.83±0.67 | 2.82±0.64 | 0.960 | 0.988 |
| T5 | 2.46±0.49 | 2.59±0.24 | 0.613 | 0.983 | 2.45±0.45 | 2.51±0.53 | 0.692 | 0.988 |
| T6 | 2.29±0.41 | 2.33±0.13 | 0.827 | 0.983 | 2.30±0.38 | 2.28±0.42 | 0.880 | 0.988 |
| T7 | 2.21±0.37 | 2.28±0.09 | 0.716 | 0.983 | 2.23±0.35 | 2.18±0.38 | 0.675 | 0.988 |
| T8 | 2.18±0.40 | 2.24±0.12 | 0.744 | 0.983 | 2.20±0.39 | 2.15±0.39 | 0.657 | 0.988 |
| T9 | 2.23±0.39 | 2.30±0.05 | 0.747 | 0.983 | 2.24±0.36 | 2.23±0.41 | 0.946 | 0.988 |
| T10 | 2.25±0.46 | 2.32±0.13 | 0.754 | 0.983 | 2.25±0.38 | 2.26±0.53 | 0.934 | 0.988 |
| T11 | 2.29±0.46 | 2.30±0.14 | 0.983 | 0.983 | 2.29±0.40 | 2.29±0.54 | 0.988 | 0.988 |
| T12 | 2.39±0.48 | 2.36±0.06 | 0.926 | 0.983 | 2.35±0.36 | 2.49±0.69 | 0.452 | 0.988 |
| Abbreviations: HI, Haller index; SD, standard deviation; BH q values were calculated separately across T3-T12 within each outcome using the Benjamini-Hochberg procedure; *q<0.05. | | | | | | | | |

**Table S38 Individual discriminatory performance of adipokines associated with neurological outcome after FDR correction**

| Adipokine | AUC (95% CI) |
| --- | --- |
| CTRP6 | 0.812 (0.698–0.926) |
| Chemerin^†^ | 0.719 (0.591–0.846) |
| Omentin-1 | 0.713 (0.577–0.848) |
| CTRP9 | 0.688 (0.548–0.827) |
| Vaspin | 0.683 (0.552–0.815) |

Note: ^†^For Chemerin, the direction of the predictor was reversed so that an AUC greater than 0.5 represented higher probability of a favorable neurological outcome with lower Chemerin concentration. AUC, area under the receiver operating characteristic curve; CI, confidence interval; CTRP, C1q/TNF-related protein; FDR, false discovery rate.

**Table S39 Complete coefficient estimates from logistic regression models for favorable neurological outcome in Cohort 2**

| Model | Variable | β | SE | OR (95% CI) | P value |
| --- | --- | --- | --- | --- | --- |
| CPR duration model | Intercept | 1.499 | 0.525 | 4.479 (1.600–12.538) | 0.004 |
|  | CPR duration, per min | −0.146 | 0.037 | 0.865 (0.803–0.930) | <0.001 |
| Clinical model | Intercept | 2.773 | 0.870 | 16.013 (2.910–88.123) | 0.001 |
|  | CPR duration, per min | −0.131 | 0.039 | 0.877 (0.813–0.946) | <0.001 |
|  | Lactate, per mmol/L | −0.203 | 0.101 | 0.816 (0.669–0.996) | 0.045 |
| Extended model | Intercept | −4.972 | 2.497 | 0.007 (<0.001–0.926) | 0.047 |
|  | CPR duration, per min | −0.148 | 0.054 | 0.862 (0.776–0.958) | 0.006 |
|  | Lactate, per mmol/L | −0.160 | 0.123 | 0.852 (0.669–1.086) | 0.196 |
|  | CTRP6, per ng/mL | 2.184 | 0.717 | 8.880 (2.179–36.187) | 0.002 |

Note: The dependent variable was favorable neurological outcome. Odds ratios were calculated per one-unit increase in each predictor. CPR duration was measured in minutes, lactate in mmol/L, and CTRP6 in ng/mL. The clinical model included CPR duration and lactate. The exploratory extended model additionally included CTRP6. The selection of CTRP6 was informed by the FDR-adjusted adipokine comparisons and individual discriminatory performance and was not prespecified.

**Table S40. Firth logistic regression sensitivity analysis of the exploratory extended model**

| Predictor | Firth OR (95% CI) | p value |
| --- | --- | --- |
| CPR duration, per 1 min | 0.882 (0.787–0.954) | <0.001 |
| Lactate, per 1 mmol/L | 0.880 (0.681–1.067) | 0.211 |
| CTRP6, per 1 ng/mL | 6.433 (2.120–27.636) | <0.001 |

Note: Firth bias-reduced logistic regression was performed as a sensitivity analysis because of the small number of favorable neurological outcomes.

**Table S41. Bootstrap internal validation of the exploratory prediction models**

| Model | Apparent AUC | Corrected AUC | Corrected calibration intercept | Corrected calibration slope | Corrected Brier score | Corrected Emax |
| --- | --- | --- | --- | --- | --- | --- |
| CPR duration | 0.846 | 0.843 | 0.023 | 0.979 | 0.165 | 0.094 |
| Clinical model | 0.876 | 0.868 | 0.006 | 0.941 | 0.152 | 0.101 |
| Extended model | 0.938 | 0.930 | −0.001 | 0.885 | 0.115 | 0.121 |

Note: Optimism-corrected estimates were obtained using 1,000 bootstrap resamples. Predictors were fixed within each model. The data-informed selection of CTRP6 was not repeated within the bootstrap procedure.
