# Supplementary material for: CT-derived subcutaneous adipose tissue thickness and circulating adipokines in relation to neurological outcome after in-hospital cardiac arrest: two parallel cohort analyses
Source: Front Neurol. 2026 Oct 20;17:1898760. doi: 10.3389/fneur.2026.1898760 (PMC13727567; doi:10.3389/fneur.2026.1898760)
Supplement: Supplementary file 2 [file Table_2.docx]

**Supplementary file 2**

**Figure. S1. Reconstruction and measurement of the patient’s bony thorax.** (A) Front view. (B) Left side view. (C) Back view. (D) Right side view.

**Figure S2. Different posture while performing two CT scans in the same patient.** (A) pre-compression CT in supine position. (B) post-compression CT in prone position.

**Figure S3.** **Bootstrap-corrected calibration of the clinical and extended models for favorable neurological outcome in Cohort 2.** (A) The clinical model included CPR duration and lactate. (B) The exploratory extended model included CPR duration, lactate, and CTRP6. Calibration was assessed using 1,000 bootstrap resamples. The diagonal line represents perfect agreement between predicted probabilities and observed outcome proportions. Each model was refitted using its fixed set of predictors in the bootstrap samples; the data-informed selection of CTRP6 was not repeated within the bootstrap procedure.

**Figure S4. Two patients were randomly selected and their bony thorax were reconstructed, the horizontal line indicated the fourth intercostal plane.** (A). 46, M, CA of cardiac origin, the forth intercostal plane were at the same level of the sixth vertebral plane, discharged with good neurological outcome; (B). 23Y, F, CA of non-cardiac origin, discharged with unfavorable neurological outcome the forth intercostal plane were at the same level of the nineth vertebral plane
